# A review and perspective of existing research on the release of nanomaterials from solid nanocomposites

**DOI:** 10.1186/1743-8977-11-17

**Published:** 2014-04-07

**Authors:** Stephan J Froggett, Shaun F Clancy, Darrell R Boverhof, Richard A Canady

**Affiliations:** 1 Froggett & Associates LLC, 3417 Evanston Ave N. Suite 502, Seattle WA, USA; 2 Evonik Corporation, 299 Jefferson Road, Parsippany, NJ, USA; 3 The Dow Chemical Company, 1803 Building, Midland, MI, USA; 4 ILSI Research Foundation, Center for Risk Science Innovation and Application, 1156 Fifteenth St. NW, Suite 200 Washington, DC, USA

**Keywords:** Nanomaterial release, Nanocomposite, Consumer products, Exposure, Nanotechnology, Release methodology

## Abstract

Advances in adding nanomaterials to various matrices have occurred in tandem with the identification of potential hazards associated with exposure to pure forms of nanomaterials. We searched multiple research publication databases and found that, relative to data generated on potential nanomaterial hazards or exposures, very little attention has focused on understanding the potential and conditions for release of nanomaterials from nanocomposites. However, as a prerequisite to exposure studying release is necessary to inform risk assessments. We identified fifty-four studies that specifically investigated the release of nanomaterials, and review them in the following release scenario groupings: machining, weathering, washing, contact and incineration. While all of the identified studies provided useful information, only half were controlled experiments. Based on these data, the debris released from solid, non-food nanocomposites contains in varying frequencies, a mixture of four types of debris. Most frequently identified are (1) particles of matrix alone, and slightly less often, the (2) matrix particles exhibit the nanomaterial partially or fully embedded; far less frequently is (3) the added nanomaterial entirely dissociated from the matrix identified: and most rare are (4) dissolved ionic forms of the added nanomaterial. The occurrence of specific debris types appeared to be dependent on the specific release scenario and environment. These data highlight that release from nanocomposites can take multiple forms and that additional research and guidance would be beneficial, allowing for more consistent characterization of the release potential of nanomaterials. In addition, these data support calls for method validation and standardization, as well as understanding how laboratory release scenarios relate to real-world conditions. Importantly, as risk is considered to be a function of the inherent hazards of a substance and the actual potential for exposure, data on nanomaterial release dynamics and debris composition from commercially relevant nanocomposites are a valuable starting point for consideration in fate and transport modeling, exposure assessment, and risk assessment frameworks for nanomaterials.

## Review

### Introduction

**Figure 1:**
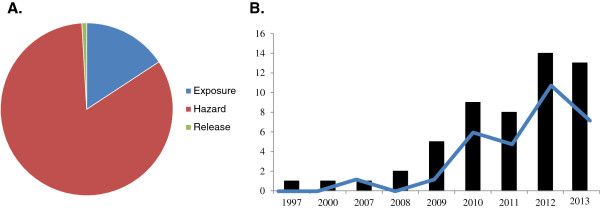
**Published literature on release form nanocomposites.** The “nanorelease” picture in terms of how many research articles have been published. **(A)** Using the ICON online database of nanotechnology environmental health and safety research, we report the number of articles identified by the “exposure” and “hazard” search terms, and compare these to the release studies we identified through multiple search engines. Considerable attention has been directed toward examining intrinsic hazards (83%) of nanomaterials, and less on potential exposure (16%) and least on release from nanocomposite (0.8%). **(B)** Since the first nanorelease study we identified in 1997, understanding release from solid, non-food nanocomposites has received increasing attention (bars) and an increasing number of these studies have been rigorous experiments (line).

Solid nanocomposites [1], made by combining conventional composite matrices with nanoparticulate additives, have been demonstrated to exhibit material properties superior to those of conventional composite (e.g. [2-4]). While nanocomposites of either carbon nanotubes (CNTs) or silica-nanoparticles (silica-NPs) embedded within thermoplastic polymers are common in the research literature, commercial availability of these and other nanocomposites is relatively new to the marketplace. Nonetheless, production volumes are increasing [5,6], as is the manufacturing base, which together are resulting in sizeable market sales [7]. In tandem with the realization of the benefits of nanocomposites, significant attention has focused on identifying potential hazards intrinsic to dissociated nanomaterials (e.g. [8-10]), potential exposure (e.g. [11,12]) and potential release pathways (e.g. [13]). Examination of the International Council of Nanotechnology (ICON) environmental and health literature database indicates that 83% of research focuses on nanomaterial hazard and 16% on potential exposure, while less then 1% on the release of nanomaterials from nanocomposites (Figure 1A). The research on intrinsic hazards of dissociated nanomaterials, and potential for exposure in the workplace have raised awareness to occupational safety needs when handling discreet nanomaterials [14,15], and led to investigation of potential workplace releases during industrial machining of nanocomposites [16].

Here, we were motivated to review the literature with a focus on understanding what is known about releases from solid, non-food commercial nanocomposites during normal uses, disposal, or recycling – conditions with a far larger exposure potential for consumers and the environment. Increasingly, consumers and the environment will interact with nanocomposites, from which the added nanomaterials must first be released before any exposure can occur. However, there is a paucity of data on release of nanomaterials from solid nanocomposites under these scenarios (Figure 1A). While some work has highlighted the possibility of release from nanocomposites [17], further study and methodological harmonization is needed [18,19]. An inability to compare results across studies has, in part, fostered the continued development of consumer and environmental exposure models based on the assumption that all nanomaterials will be released from nanocomposites in their dissociated form [20]. With increasing attention on release from nanocomposites (Figure 1B), it is becoming increasingly apparent that the assumption that discrete nanomaterials will be released is generally not accurate [21]. To facilitate broader understanding of the conditions when release from consumer nanocomposites may occur, the composition of the released debris, and the challenges that exist for such investigations, we offer this review. We believe that robust data on release from such nanocomposites are necessary to make accurate predictions about common scenarios that may induce release across widespread consumer and professional uses.

### Literature search approach

In 2013, we conducted a search of two large and publically available research publication databases, PubMed (NCBI) and Chemical Abstracts (CAS/STN) using three broad search terms: nanoparticle, nanomaterial and release. This key word search identified over 10,000 articles. However upon review, only fifty-four studies describe research efforts that deliberately investigate release from a solid, non-food nanocomposite. We review these ‘nanorelease’ studies grouped by the methods used to induce release. While hazard is a key component in the determination of risk, here we place specific emphasis on the release potential – the possibility that a nanomaterial added to form a nanocomposite may become separated from that composite matrix and released into the surrounding environment. Understanding release potential is an important starting point towards accurately understanding exposure potential.

### Release scenarios

**Figure 2:**
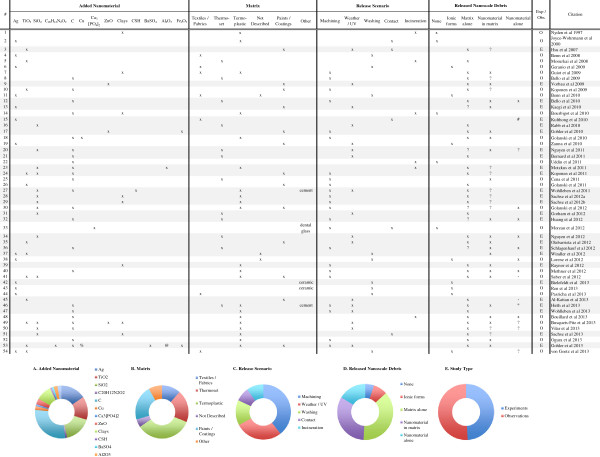
**Overall summary of the reviewed nanorelease studies.** Several key details about all fifty-four nanorelease studies we identified and discuss in this review are presented in the table. While the experimental studies are highlighted, all provide information about which nanomaterials have been added to different base matrices and examined under the various release scenarios. The debris released is most often a mixture of multiple types, particle of matrix alone, particles of matrix with the nanomaterial embedded and the nanomaterial fully dissociated from the matrix. In addition, a few studies did not identify any release, or clearly identified the release of ionic forms of the added nanomaterial, but not the nanomaterial itself. Across study summaries are present in charts: **(A)** the added nanomaterial, **(B)** the matrix, **(C)** the release scenario examined, **(D)** the released debris identified and **(E)** the number of experiments versus observational studies. (#) Authors used GFAAS, which incinerates the matrix in the process of identifying chemical composition. Thus we are unable to determine if the nanomaterial was bound to, or dissociated from the matrix; (*) Authors report release only after a combination of weathering and machining; (-) authors report insignificant but detectable levels of dissociated nanomaterial; (?) data supporting this result are indirect or not presented, but described by the authors; (exp) rigorous experiments with replicate testing and negative controls (samples of matrix without added nanomaterial) examined; (obs) observational studies with no control samples and/or not replicate testing; (C) refers to any one of multiple forms of Carbonaceous nanomaterials, including: single and multi-walled carbon nanotubes, graphene oxide, carbon black and uncharacterized carbon nanotubes; (CSH) are calcium silicate hydrates; (%) a complex copper II with chlorinated phthalocyanine; (@) alumina based Cobalt Blue.

Through consumer product use, disposal or recycling, nanocomposites encounter potentially degrading mechanical, thermal and/or chemical energy inputs that may result in the release of the embedded nanomaterials. However, most of the nanorelease literature focuses on scenarios where multiple input energies are present, with a high potential for release. Thus we have organized our review around the following scenarios: machining, weathering, washing, contact and incineration; presenting them from most to least well examined. Within the machining, weathering, washing and contact scenarios, we have sub-divided these scenarios to discuss similar methodologies together. For each study we review the methods used to induce release, detect, measure and characterize the released debris, the findings and, whenever possible, the composition of the nanocomposite. Although we highlight the experimental studies, given the dearth of data at this time, we believe the results from both observations and experiments provide potentially valuable insights into release dynamics. Even, within this limited data set there much information that should serve as a useful benchmark for the state of knowledge on the release of nanomaterials from nanocomposites (Figure 2).

### Machining scenarios

Improving the durability of composites has been the focus on ongoing research for decades (e.g. [22-24]) and by the end of the millennium, investigators had begun to evaluate potential benefits from adding nanomaterials to conventional composites (e.g. [2-4]). Accordingly, many of the same methodologies, including standardized protocols which were used to demonstrate composite durability, were applied to nanocomposites. As interest shifted from showing nanocomposite durability, to examining release, many of the same methods began to be used in this novel context. As such, many of the early nanorelease studies report experimental design challenges as much as the released debris. In this section, we review studies employing a wide range of machining instruments and methods used to apply mechanical forces to nanocomposites. The non-standardized and typically hand-operated methods include, cutting, grinding, shredding, sanding, and drilling. In addition, the Taber Abraser, with its many standardized abrasive wheels and protocols has also been used extensively. We close this section with a few novel machining scenarios; one that simulates the conditions in the oral cavity to examine release from dental nanocomposites, and a pair of studies that combine weathering and then apply machining.

### Cutting

Bello and colleagues [25] are the first to present the challenges of measuring release during nanocomposite cutting in a laboratory setting. During their observational study, two types of lab-made nanocomposites were tested, an epoxy resin layer laminate and an alumina fiber cloth. For each type, a control composite matrix - with no added nanomaterial was also included for comparison to the nanocomposites, both of which contained CNTs. Multiple samples of variable thicknesses were made and machine cut with either a dry band-saw or a wet rotary cutting wheel. Release measurements were taken over the course of 3 – 5 cuts per sample, on three separate occasions over the course of a year. The authors report, regardless of the composite type and the presence or absence of CNTs, released ultra-fine particles were always detected, and correlate increases in ultra-fine particle release to matrix thickness. The four-ply (2.9 mm thick) base-alumina composite released the most, while the 1-ply (0.6 mm thick) CNT-alumina composite generated the least. Focusing on the affects of machining, dry-cutting either the controls or the nanocomposites, with the band-saw produced relatively more ultra-fine particles then wet-cutting. Aerosolized particles during dry cutting were 71-89% in the 1- 10 μm range, 6-25% in the 0.1-1 μm range and only 1-10% in the <100 nm size range. Subsequent transmission electron microscope (TEM) and scanning electron microscope (SEM) analysis revealed numerous sub-micron sized fibers, but no clearly identifiable CNTs or bundles were observed.

In another workplace setting, a four day occupational safety study conducted by National Institute for Occupational Safety and Health (NIOSH), Methner et al. [26] investigated release of carbon nanofibers (CNFs) from epoxy based nanocomposites during three machining processes: wet saw cutting, grinding and sanding (by machine and by hand). The authors directly monitored released debris concentration and particle size at two sites (when possible), the processing area and in the operator’s breathing zone, by Condensation Particle Counter (CPC), HHPC-6 and DustTrak DRX Aerosol Monitor. Alongside the direct air sampling, debris samples were collected on a filter for subsequent characterization under TEM. The nanocomposite used in this study is a proprietary compound of CNF and epoxy, which is purportedly common in the aerospace industry. During wet saw cutting, direct measurements were not made in the processing area, but nanoscale debris was detected in the operator’s breathing zone. Subsequent TEM analysis of debris collected at both sites indicated the presence of CNFs both dissociated from, and bound to the matrix. In contrast, no nanoscale debris was detected at either sampling site during either machine or hand sanding. However, under TEM, dissociated and matrix bound CNFs were identified in the processing area during machine sanding, but not in the breathing area. And finally, during hand sanding, TEM images reveal matrix bound CNFs only. Based on these data, the authors suggest that sanding either results in the release of larger scale debris that often contain CNFs, or that the direct sampling methods were not entirely accurate. Nonetheless, it appears clear that all of the machining processes tested induce release and that workers’ should utilize personal protective devises to reduce potential exposure, as has been previously recommended [15,16].

### Grinding

Methner et al. [26] and Ogura et al. [27] report the only investigations of release due to grinding lab made nanocomposites. Methner et al. [26], using the same thermoset/CNF nanocomposite described earlier, report detecting nanoscale debris by direct measurements at both sites, and under TEM identifying bound and dissociated CNFs as a result of grinding. In contrast, Ogura et al. [27] examine a thermoplastic/CNT (5% by weight (wt)) nanocomposite and a control matrix with no added CNTs. Based on direct measurements by CPC, the authors report detecting considerable release of nano-scale particles from both the nanocomposite and the control matrix. SEM analysis of the released debris revealed no dissociated CNTs, rather micron and nanoscale particles of matrix with CNTs protruding from the surface. While these data are in contrast to those reported by Methner et al. [26], the lack of a description of the nanocomposites in both studies, and the absence of a description of the grinding parameters (e.g. force exerted, duration) prevent more rigorous comparisons being made.

### Shredding

To our knowledge, Raynor et al. [28] report the only investigation of shredding induced release, a likely scenario for the recycling of nanocomposites. The nanocomposite used in this experiment is a thermoplastic with 5% wt montmorllonite nanoclay, and is representative of the type of nanocomposites being increasingly used by the automobile industry. As the authors highlight, with 15 million vehicles in the U.S. and 8–9 million cars in the E.U. being recycled annually, the potential exposure to released nanomaterials during this process could be considerable for recycling facility employees, area residents and the surrounding environment. The authors investigated release from a nanocomposite test plate of 18CPP091/nanoclay and two negative controls, a conventional talc composite and a test plate comprised of the base polypropylene matrix alone. The authors used an encased 3.7 kW throat granulator to shred a total of 480 test plates, once every 15 sec, during each two-hour testing period. Through a metal duct connected to the experiment enclosure, the authors made direct measurements of released particle number concentration (via P-Trak), mass concentration (via DustTrak) surface area concentration (via AeroTrak) and size distribution (via a fast mobility particle scanner (FMPS) and an optical particle counter (OPC)), and further characterized released debris under SEM. The authors report significant release of nanoscale debris from all of the test plates during shredding, and note that the particle concentrations were highest from the matrix alone and least from the talc composite. The nanocomposite plates are reported to release a mid-level concentration of nanoscale particles. Under SEM, the authors were unable to identify any nanoclay particles dissociated from the matrix, and in debris less then 100 nm in diameter, no nanoclay particles were detected.

### Sanding

Koponen and colleagues [29] conducted one of the first controlled, bench-top machining experiments of nanocomposite coatings. The authors received thirteen coated medium density fiberboard (MDF) “test plates” from the Danish paint industry and present data from three plates, one control and two test plates coated with two different nanocomposite paints. All of the coatings were pre-painted onto the test plates were identified as: a control paint with no added nanomaterial, a paint containing 95 nm carbon black, and a paint with 17 nm titania nanoparticles (titania-NPs) added. No additional, presumably proprietary, details regarding the added NPs are provided, which is common for nanorelease studies using commercially relevant nanocomposites. In this experiment, the authors refined a containment chamber shown to reduce workplace “dust” [30-32] such that released particle concentration and size could be measured as a hand-held orbital sander was used to abrade the control and test coatings on the MDF plates. The released debris emitted from the test coating and the exhaust from the sander were both measured with an aerosol particle sizer (APS) and FMPS. The authors report sanding resulted in the release of nanoscale and ultra-fine particles from each test plate, including the control. Using diameter size to characterize the released particles, the authors fitted the data to a five modal distribution curve, and show the smallest two peaks were < 20 nm, the middle peak was about 200 nm and the largest two were 1 and 2 μm. Based on a previous report [33] and on the results collected during this study, the authors attribute the smallest two modes of released particles to the orbital sander’s electrical motor, while the mid-sized mode (~200 nm) comprised a combination of electrical motor “spark-particles” and matrix debris. The authors report the largest two modes contain mostly nanocomposite debris, but were unable to identify individual nanoparticles of either titania or carbon black.

In follow up study, Koponen et al. [34] again investigated paints and a lacquer with added nanomaterials applied to MDF plates, supplied by industry partners. As in their earlier study, the authors report the sander motor as a significant source of aerosolized particles <50 nm, while the dominant released debris size ranged from 100-300 nm regardless of the coating being sanding. The authors were unable to identify any clear differences in terms of release rates or released particle size between paints with or without added nanomaterials. The authors do note however, that the pressure applied by the hand held sander and the grit size of the sandpaper could, at least in theory result in different release dynamics. In addition, the authors note the limitations of APS and FMPS, and suggest subsequent characterization of released particles under SEM would be helpful in clearly distinguishing discrete nanoparticles from nano-sized agglomerates and matrix embedded nanomaterials. In this observational study, the authors report they assume that no discrete nanomaterials were released due to sanding and plan further analysis. Under identical experimental conditions and using the same detection and characterization methods, Saber et al. [35] report similar results for an even broader range of reference coatings and nanocomposite paints, lacquers and binders then previously tested. In each of these studies, no systematic differences were identified in the released debris number or size, and that the added nanoparticles were retained within the matrix or particle aggregates [29,34,35].

In an effort to more accurately model professional sanding forces, Göhler and colleagues [36] used a Dremel tool, instead of a hand-held orbital sander, in an effort to develop a more commercially relevant experimental approach. The authors compared multiple samples, including control coatings with no added nanomaterial. Test samples included, zinc oxide nanoparticles (zinc oxide-NP) added to a two-pack polyurethane, applied to a steel plate, and separately were added to a white-pigmented architectural coating and applied to a fiber cement plate. In addition, iron oxide nanoparticles (iron oxide-NP) were added to the white-pigmented architectural coating and applied to a fiber cement plate. Control coatings, with no added nanomaterials were made with both, the polyurethane and the architectural coating, and applied to the steel plate and the fiber cement plate, respectively. The authors report that 75% of the added zinc oxide-NP were <100 nm, while only 25% of the added iron oxide-NP were less than <100 nm. The authors used FMPS along with a laser aerosol particle (LAP) size spectrometer, and a CPC to determine the number of particles released during sanding. The authors report machining generated nanoscale debris was released from all of the coatings tested, including the controls. Based on TEM analysis, the authors clearly demonstrate that both iron oxide-NP and zinc oxide-NP were embedded within the released debris, and report being unable to detect any dissociated, discrete iron oxide-NP or zinc oxide-NP.

Both Wohlleben et al. [37] and Cena and Peters [38]) used sandpaper to induce release, but differences in the nanocomposites and methods used direct comparisons should be made cautiously. Wohlleben et al. [37] performed controlled experiments in triplicate to investigate release from both cementitious and thermoplastic nanocomposites containing CNTs, and a polyamide (PA) embedded with amorphous silica nanoparticles (silica-NP). Results from sanding these nanocomposites were compared to control composites containing the base materials, but no added nanomaterial. Their experiment was conducted in a confined apparatus supplied with filtered air, and used KK114F sandpaper (grit size P320) with constant pressure (9.81 N) and velocity (6.40 m/s) on all of the samples. Any released debris was captured 1 cm above the sample. Consistent with previous machining experiments [29,36], the authors observed nanoscale debris released from all of the samples tested, regardless of the presence or absence of added nanomaterial. Further, by scanning mobility particle sizer (SMPS), the authors identified a significant volume of particles less than 100 nm that were released from both the control and nanocomposite cement samples. The particles released from both of the thermoplastic samples however, were reported to be larger, around 2 μm. Follow-up SEM observations of the all released debris from the nanocomposites and the control composites did not reveal any CNTs dissociated from the composite matrix. In a more recent experiment Wohlleben et al. [39], employing the same methods [37], sanded a relatively soft thermoplastic with 3% wt CNTs (TPU/CNT). Similar results are reported, nanoscale debris was released from the reference matrix (without CNTs) and the nanocomposite, CNT protrusions were identified on the nanocomposite surface but no dissociated CNTs were identified among the debris.

Cena and Peters [38]) used a different sandpaper and grit size (3 M® 220 grit) to abrade nanocomposite sticks of epoxy with 2% by weight multi-walled carbon nanotubes (MWCNTs), but report no controls. For this observational study, an operator manually sanded the test sticks while a CPC and an Optical Particle Counter (OPC) were used to measure aerosol concentrations for 15–30 min in two locations, adjacent to the sanding and within the operator’s breathing zone. In contrast to previous [29,36,37] and subsequent [26] findings, the authors report no significant difference in the concentration of released nanoscale debris at either sampling location, and that these concentrations were insignificantly different from background levels. Without subsequent analysis, these data alone would suggest no release occurred under these conditions. However under TEM, the authors were able to observe irregularly shaped particles >300 nm, some with protrusions they described as resembling MWCNTs, and observed later by Hirth et al. [40]. No dissociated MWCNTs were identified however.

In a more recent experiment Huang et al. [41] used similar epoxy test sticks, but evaluated a wider concentration range (1 - 4% by wt) of added MWCNTs, in addition to control sticks with no added nanomaterial. In contrast to the Cena and Peters [38]) study, the authors used a medium grit disc sander to abrade the test sticks. Under these conditions, the release of nanoscale debris was detected from all of the test sticks, including the control. The authors describe a bi-modal distribution of released debris size, either < 100 nm or > 500 nm. Under TEM analysis, the authors reported observing MWCNTs protruding from the debris released from each of the nanocomposite sticks (1-4%), but not from the control stick. In addition, the authors identified free, dissociated MWCNTs among the released debris. At this time, these data, along with those from Schlagenhauf et al. [42] discussed below, are the only machining data from controlled experiments that demonstrate the release of nanomaterial dissociated from the nanocomposite matrix.

### Drilling

We are aware of only three, two nearly identical studies by Sachse et al. [43,44] and one one by Bello et al. [45] that describe release due to drilling nanocomposites. In both Sachse et al. [43,44] experiments, the authors used a hand held Makita angle drill, situated outside of an experiment enclosure to reduce confounding spark emissions reported by others [29,33,34] from the drill motor. The authors used a combination of a CPC and SMPS to measure aerosolized particles, and subsequently characterized the released debris under SEM with EDX. Both studies tested the base matrix, PA-6 alone as a negative control, and performed their experiments in triplicate. The key difference between the two studies is the lab made nanocomposites investigated. In one study [43], the authors report investigating two nanocomposites, 5% MMT without modification and 5% wt silica-NPs, and two micro-composites containing foam-glass-crystals and glass fibers. In the second study, Sachse et al. [44] examined a single nanocomposite, PA-6 with 5% wt organically modified montmorillonite (MMT) nanoclay added. During these drilling experiments, both PA-6/MMT nanocomposites released the lowest concentration of nanoscale particles, fewer then the PA-6 neat matrix [43,44]. In Sachse et al. [43] the layered MMT nanocomposite released 1.5 times less then the PA-6 composite, while in Sachse et al. [44] the authors report the organically modified MMT nanocomposite released 20 times less then the PA-6 control composite. In contrast, the silica-NP nanocomposite released 56 times more nanoscale particles compared to the PA-6 composite [43]. In neither study do the authors report identifying the added nanomaterial dissociated from the matrix among the released debris.

In contrast, Bello et al. [45] report identifying bundled CNTs, among the release debris generated by drilling. Using the same CNT-alumina and CNT-carbon nanocomposites, along with similar detection and characterization methods as in their previous, cutting study [25], the authors report significant differences in release dynamics and debris composition in response to drilling [45]. In this rigorous experiment, high speed dry drilling was shown to generate more overall release but fewer respirable fibers than cutting. However, among the release debris were aggregates of CNTs, which were previously not observed when cutting the same nanocomposites. In addition, the authors report nanocomposite thickness did not influence release when drilling, as it did during cutting. This study clearly demonstrates differences in release dynamics and particle composition due to the machining process rather then the nanocomposite. The work highlights a need to consider both the degradation mechanism and the nanocomposite as both may affect release.

### Standardized machining methods

In contrast to the operator-handled machining methods discussed thus far, the following machining studies used a Taber Abraser to wear the surface of test samples [37,39,42,46-50]. The Taber Abraser, a precision-built instrument that has been used since the 1930’s, enables accelerated wear testing on rigid and flexible materials ([51,52]. Although several standardized methods exist, its use in the context of inducing release debris is relatively novel, and how this wear relates to real-world conditions is unknown. Two early studies adapted the standardized methods by enclosing the abraser apparatus to capture released wear debris from a nanocomposite coating [50] and a fabric [49]. Vorbau et al. [50] performed a machining experiment in triplicate with three commercially available nanocomposite coatings: (1) a two-pack polyurethane coating with up to 6% zinc oxide-NP applied three times to a steel plate, and separately to an oak veneer fiberboard, (2) a UV curable clear coat with up to 3% zinc oxide-NP applied in three layers to oak veneer fiberboard, and (3) a white pigmented architectural coating with up to 5% zinc oxide-NP applied in two layers to fiber cement boards. In addition, control samples of each coating type were prepared with no added nanomaterial. In contrast, Guiot et al. [49] do not describe control samples for two of the three nanocomposite-fabrics investigated, and do not discuss repeated testing. Thus, the authors describe observational data from three nanocomposite fabrics: (1) a cotton fabric coated with 20 nm silica nanoparticles (silica-NP) deposited by colloidal suspension; (2) a cotton fabric coated with polystyrene-latex nanoparticles (40-100 nm) and, (3) an “advanced fabric” with a PET layer coated with an additional PVC layer containing nano-clays. Only this, “advanced fabric” has a control sample with no added nano-clay for comparison.

Both Vorbau et al. [50] and Guiot et al. [49] used SMPS to measure particle size distribution and a CPC to measure the number of particles released. Both of these instruments were attached to a low volume-sampling hood that encased the Taber Abraser to capture aerosolized debris released from the test coatings and fabrics. Under these conditions, released nanoscale debris was detected from all of the samples investigated, including the controls with no added nanomaterial. Guiot et al. [49] then compared the released particle size distribution curves from the advanced nano-clay fabric to the control with no nano-clay, and suggest an observed peak at 50 nm in size represents the added nano-clay. However, the authors do not describe any additional evidence in support. In contrast, Vorbau et al. [50] used transmission electron microscopy (TEM) to observe the released debris. Based on the TEM analysis, the authors report some of the released debris contain partially embedded zinc oxide-NP, but no dissociated, discrete zinc oxide-NPs were identified.

Subsequent studies have used the Taber Abraser to investigate release from a range of nanocomposites under multiple conditions [46-48]. In an initial observational study [46], thermoplastic nanocomposites of either poly-methyl methacrylate (PMMA) with 10% copper nanoparticles (copper-NP) or polycarbonate (PC) with 3% carbon nanotubes (unspecified CNTs) were abraded with a range of tools, including a Taber Abraser, a stainless steel brush and abrasive ribbons of SiC [53]. Using an electrical low-pressure impactor (ELPI) and a nanometer aerosol sampler (NAS) to detect particles released from the nanocomposites during each of the machining methods. Both nanocomposites released nanoscale debris as a result of machining, but no discrete copper-NPs or CNTs were observed among the released debris under TEM or SEM analysis. The authors do report observing an increase in nanoscale debris as machining speed, or applied force was increased. In addition, the authors report that the grit-size of the abrasive material used influenced the proportion of debris in the nanoscale fraction, and that the stainless steel brush generated the largest volume of nanoscale debris.

In a follow up study with controls, but no replicates, Golanski et al. [47] compared two machining scenarios, wet and dry. The Taber Abraser was used to conduct “dry” machining, while an Elcometer 1720, was used to perform “wet” machining. Both approaches were used to investigate release from four paints. Two paints were nanocomposites and two contained no added nanomaterial. The two nanocomposite paints contained titania-NPs, one with calcium carbonate, and the other with pigment-grade titania but no calcium carbonate. The paints without nanomaterials were both made with calcium carbonate and either pigment-grade titania or no titania at all. All four paints were applied to polyvinyl chloride (PVC) or glass substrates with a dried film thickness of about 150 μm. The researchers used an ELPI and a laser granulometer to measure released debris size from each of the paints under wet and dry machining conditions. These approaches detected the release of micronic and smaller particles, but no nanoscale particles appear to have been released from any of the four paints abraded under either condition. Follow up SEM analysis of the released debris from the nanocomposite paints, under both wet and dry conditions, revealed titania-NPs embedded within the released particles but no free or agglomerated titania-NP dissociated from the matrix.

Wohlleben et al. [37], as part of a larger experiment with the same set of thermoplastic (PA and POM) and cementitious nanocomposites described earlier, also used the Taber Abraser to investigate release. Similar to the sandpaper results, no significant differences in release debris size or concentration was observed by SMPS among all of the nanocomposites and their control composites. Further characterization of the released debris by SEM or TEM was not performed. In a recent experiment, Wohlleben et al. [38] investigated release from a relatively flexible nanocomposite made from thermoplastic polyurethane (TPU) with CNTs. The nanocomposite was exposed to mechanical stress from the Taber Abraser, in addition to sanding and weathering (discussed previously and in weathering) and released debris particle size was measured and characterized under SEM. The authors report a peak released particle size of 30 nm, which may include dissociated CNTs below the limit of detection. However, subsequent analysis of SEM and TEM images did not reveal any dissociated CNTs among the released debris.

In contrast to the machining studies previously discussed, one observational study ([48];) and two controlled experiments [41,42] report identifying dissociated nanomaterials among the released debris from nanocomposites. Golanski et al. [48] investigated release from two thermoplastic-CNT nanocomposites, and one thermoset (epoxy) - CNT nanocomposite, in response to multiple abrasive forces. The authors describe three nanocomposites, two thermoplastics, PA with 4% CNT and PC with 4% CNT, and an epoxy with 0.8% CNT. It appears Golanski et al. [48] report results similar to those reported earlier Golanski et al. [46]); machining of thermoplastic nanocomposites containing CNTs with a Taber Abraser generates nanoscale release debris, but no release of the added nanomaterial. And although control composites (without added CNTs) were also abraded with the Taber Abraser, it does not appear they were subjected to the same treatment – receiving only “low machining” instead of the “high machining” performed on the nanocomposites. Thus, the logical suggestion made by Golanski et al. [48], that increasing machining intensity results in an increase in the release of nanoscale debris, is not necessarily supported by the data reported here, or in their previous study [46], where no controls are described. However, the authors also describe identifying silica-NP and unspecified nanoparticles from previously unmentioned PVC fabrics among the release debris. While the silica-NP may be an artifact from the abrasive wheels of the Taber Abraser, we are uncertain how to interpret the identification of uncharacterized nanoparticles of unknown origin. In addition to these data, Golanski et al. [48] describe using two other tools (a steel rake and an engraver) in an attempt to dislodge CNTs from the nanocomposites investigated. It’s unclear if the ‘steel rake’ is the same as the ‘steel brush’ described by Golanski et al. [46], and reported to be ten times more effective at inducing release by Tardiff [54]. In Golanski et al. [48] the rake is clearly shown attached to a Taber Abraser, but later the authors describe the operator “feels” the vibrations generated as the rake is dragged across a nanocomposite fabric, implying the rake is a hand-held tool. Nonetheless, the authors go on to state that the rake could be used to release dissociated CNTs, but only from nanocomposites with poor dispersion. While this is an intriguing and possibly accurate assertion, it does not appear to be supported by the data presented.

Schlagenhauf et al. [42] describe a set of machining experiments with thermoset-MWCNT (0.1 - 1% by weight) nanocomposites and a control composite (0% MWCNT). In this study, released particle size was measured by APS, SMPS, FMPS and CPC, and follow up released debris analysis was performed under SEM and TEM. Based on the data collected during three separate machining measurements, the authors report that all samples tested (nanocomposites and controls) released debris with four size modes, but that none of these were less then 100 nm. The authors’ note comparing the distribution of released debris sizes from the control composite to the nanocomposites reveals a shift of 70-90 nm in the released particle size distribution. In this study, the abraded debris from the nanocomposite was larger then the released debris from the composite with no added nanomaterial. The authors also examined the release debris under TEM, and are the first to identify both free CNTs and agglomerates among the debris from the 1% CNT-nanocomposite. But no dissociated CNTs were found among the debris from the 0.1% CNT-nanocomposite or control composite. To our knowledge this and Huang et al. [41] are the only experiments demonstrating machining alone can induce the release of an added nanomaterial dissociated from the composite material.

### A novel dental machining study

A unique machining study warrants brief discussion, Moreau et al. [55] report investigating release from nanocomposite dental fillings. The authors evaluated four different fillings: a control with 0% calcium phosphate-NP (CaP-NP) + 75% glass; and nanocomposites with 10% CaP-NP + 65% glass; 15% CaP-NP + 60% glass; and 20% CaP-NP + 50% glass. Both CaP-NP and barium boroaluminosilicate glass particles with a resin of bisphenol glycidyl dimethacrylate and triethylene glycol dimethacrylate at 1:1 mass ratio were rendered light-curable with 0.2% camphorquinone and 0.8% ethyl 4-N, N-dimethylaminobenzoate to achieve Ca^2+^ and PO_4_ release and load-bearing ability. The samples were exposed to a simulated oral cavity environment via a four-station wear apparatus that used water-PMMA bead slurry reaching a max load of 76 N. The authors describe “dimple-like” wear scars on the surface of the nanocomposites as a result of the simulated wear. During the testing process, the slurry temperature was cycled from 5 - 60°C by 15 second water bath immersion. As a last step, the samples were immersed in distilled water at 37°C from 1 day to 24 months prior to analysis. The authors report that CaP-NP nanocomposite dental fillings did not degrade significantly in terms of the flexural strength or elastic modulus compared to the composites with no added nanomaterial. In fact, they performed slightly better than the commercially available samples. However, it appears that increasing the CaP-NP content decreased mechanical properties and increased observable wear scaring of the nanocomposites. While these results suggest minimal release during the wear testing, the authors’ were focused on measuring the intentional release of Ca^2+^, which occurred, with the desired effect of improving adjacent tooth enamel.

### Machining plus weathering scenario

**Figure 3:**
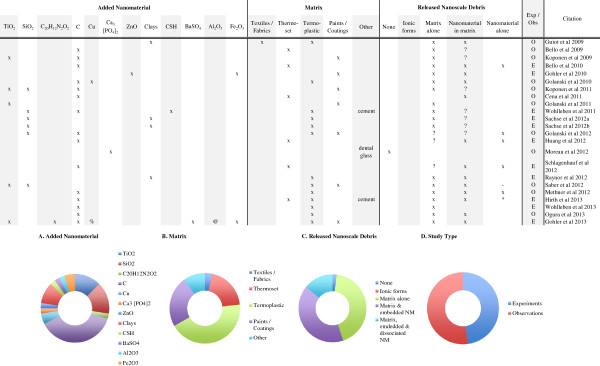
**Machining induced release from nanocomposites.** The most extensively examined release scenario, nearly half of all the nanorelease studies utilized some form of machining. All but one of these studies reports the detection of some form of release debris; most frequently (91%) are particles of matrix, and less frequently but still common (87%) are particles of matrix with embedded nanomaterials. Less then a third of the studies (30%) report identifying dissociated nanomaterials among the release debris. Across study summaries are present in charts: **(A)** the added nanomaterial, **(B)** the matrix, **(C)** the released debris identified and **(D)** the number of experiments versus observational studies. (*) Authors report release only after a combination of weathering and machining; (-) authors report insignificant but detectable levels of dissociated nanomaterial; (?) data supporting this result are indirect or not presented, but described by the authors; (exp) rigorous experiments with replicate testing and negative controls (samples of matrix without added nanomaterial) examined; (obs) observational studies with no control samples and/or not replicate testing; (C) refers to any one of multiple forms of Carbonaceous nanomaterials, including: single and multi-walled carbon nanotubes, graphene oxide, carbon black and uncharacterized carbon nanotubes; (CSH) are calcium silicate hydrates; (%) a complex copper II with chlorinated phthalocyanine; (@) alumina based Cobalt Blue.

With a few observational [26,35,48] and experimental [41,42,45] exceptions, the overwhelming finding has been that well prepared (e.g. surface modified, well dispersed) nanocomposites do not release discrete nanomaterials due to mechanical forces alone (Figure 3). Such findings have motivated at least a couple of investigators [19,40] to begin examining release under a combination of forces (e.g. chemical and mechanical) that more closely mimic real-world scenarios.

Göhler et al. [19] investigated two matrix types, PP and an acrylate coating, to which one of nine different nanoscale pigments were added. The authors made ten samples of each of the nine different nanocomposites and nine nano-coatings, as well as negative (matrix alone) and conventional (i.e. added pigments >100 nm) control composites. Four samples of each type were weathered for the equivalent of 2–5 years following [56]. After weathering, each nanocomposite and nano-coating was subjected to three forms of mechanical forces: sanding, dynamic friction and wind erosion. Thus, the authors could compare release due to machining from weathered and non-weather nanocomposites. During machining, the concentration and particle size of released debris were measured by CPC, APS and an engine exhaust particle sizer (EEPS). Subsequent debris characterization was performed under SEM and TEM with EDX. The authors report all of the samples tested, regardless of nano-pigment content or weathering, released nanoscale debris, but were unable to identify any of the added nano-pigments dissociated from the matrix under SEM or TEM. The authors also report differences in total particle release dependent on weathering and mechanical force applied to both the nano-coatings and nanocomposites. Nano-coatings released significantly more nanoscale debris due to sanding and dynamic friction, but not due to wind, after weathering. Meanwhile, the nanocomposites exhibited no significant change in nanoscale release due to sanding or dynamic friction after weathering. But the nanocomposites did release significantly more nanoscale debris due to wind erosion after weathering. With these results, the authors have clearly demonstrated that weathering, machining, and nanocomposite type all affect release rates, and that interactions between these factors are also important for understanding release.

In another first, Hirth et al. [40] describe a complex set of experiments that compare release of CNTs from four types of nanocomposites, one of which is first weathered and then exposed to a series of mechanical forces. All four nanocomposites, a thermoset (epoxy/CNT) as described in Huang et al. [41], a cementeous (CNT/cement) and two thermoplastic (CNT/POM and CNT/TPU) as described in Wohlleben et al. [37] are sanded, and the released debris is measured and characterized as described in Wohlleben et al. [37]. Based on multiple analyses and SEM imaging, Hirth et al. [40] report identifying matrix debris released from all of the nanocomposites, but are only able to identify CNTs protruding from the matrix debris from the epoxy/CNT and cement/CNT nanocomposites, similar to that reported by Wohlleben et al. [37] and Cena and Peters [38]. In both cases, the typical debris particle is 1 μm with at least 5-7 CNTs (10-50 nm diameter) protruding from the surface. No dissociated CNTs are identified among the released debris from any of nanocomposites, and no protrusions of CNTs are identified in the particles released from either of the thermoplastic nanocomposites. Machining alone did not appear to induce the release of CNTs from any of these nanocomposites.

However, Hirth et al. [40] were able to identify dissociated CNTs from the CNT/TPU nanocomposite after the combination of weathering, by either [57] or [58] methods, and subsequent mechanical forces. After the equivalent of nine months of weathering, the CNT/TPU nanocomposite was submerged in a surfactant solution and placed on a shaker for 24 hours, then exposed to ultrasonic agitation. The authors note, that only after the shaker and ultrasonic agitation, did the solution appear turbid and exhibit a color change. The solution with the negative control (i.e. TPU alone) turned yellowish, while the solution with the CNT/TPU turned grey/black, leading the authors to suggest release of CNTs had occurred at this stage in the experiment and not earlier. To varying degrees, all solution samples contained micron-sized debris and particles less then 150 nm, including the TPU alone with no CNTs. Dried suspensions were observed under SEM, allowing the authors to confirm the micron-sized and smaller debris comprised matrix with embedded CNTs as well as, occasional dissociated CNTs. The authors note a direct relationship between increased mechanical force on the weathered nanocomposite surface and an increased release of <150 nm particles and dissociated CNTs. The release rate and concentration of micron-sized debris did not appear to be similarly influenced by increased shear forces, and no differences in release were noted between wet and dry weathering. Based on the results form this study, the authors are the first to demonstrate that dissociated CNTs can be released by the machining of a weathered nanocomposite. These findings are in contrast to those reported for weathered coatings, where pigment grade titania [59] and nano-titania coatings [60] both released titania-NP in response to wet weathering without machining forces.

### Machining summary

A summary of the twenty-three studies examining release from nanocomposites due to a range of machining methods is presented in Figure 3. A brief review of the key data in the studies published to date show a focus on both CNTs and silica-NPs embedded within mostly, lab-made thermoplastic nanocomposites. Beyond these, nine other nanomaterials and five different matrices were examined under a range of machining release scenarios. For a majority of these investigations, researchers used hand-held equipment, but a significant number used standardized equipment and protocols. Almost all of the studies relied on real-time detection and subsequent microscopic analysis to characterize the release debris. Based on these measurements, the data are clear; matrix and matrix with embedded nanomaterials are frequently released from nanocomposites (91% and 87% respectively) in response to the machining forces applied. Of the twenty-three studies conducted to date, only four studies [26,41,42,48] report significant release of nanomaterials dissociated from the matrix tested, and in one study [35] insignificant but detectable levels were observed in response to machining forces alone. In another study, Hirth et al. [40] report dissociated nanomaterials were released by machining only after first weathering the nanocomposite.

### Weathering scenarios

Demand for more durable exterior composites and improved coatings (i.e. paints) have driven research to understand both the mechanisms of polymer degradation in response to long-term UV exposure and the impact of additives on degradation [61,62]. Since the early 20th century, the photochemical properties of titania have been known and utilized widely as an environmentally safe coloring additive for paints, plastics and paper [61]. The uses of nanoscale versions of previously used additives (e.g. titania), or of novel nanomaterials (e.g. CNTs) have shown the potential to make more vibrant, longer-lasting and even ‘self-cleaning’ exterior coatings. While these advances have both consumer and environmental benefits, important questions remain; is the degradation processes of these nanocomposites unique [63], and what, if any, impact does this have on added nanomaterial release [64]? To date, a few observational studies [65-67] and several experiments on a range of nanocomposites have been performed to examine release in response to UV exposure alone [68-72], or due to complex weather scenarios [37,60,73-75]. We have divided our review of these weathering studies into four sections, *complex weathering methods, UV exposure, standardized methods* and *aqueous weathering.*

### Complex weathering methods

To our knowledge, Hsu and Chein [73] were the first to experimentally investigate release from two coatings containing titania-NP exposed to complex, simulated weathering that included wind, UV and fluorescent light, with accompanied “mild” machining. Control samples were the substrates (wood, ceramic tile and polymer film) without a nanocomposite coating. One test sample was made with a spray coating containing 5% by weight polycrystalline anatase titania-NP in suspension, and subsequently applied to either the wood or polyethylene terephthalate (PET) polymer test plates. The second nanocomposite coating was a commercially available paint containing titania-NP that was applied to a ceramic tile. Two hour weathering tests were run twice with each of the samples to generate release data. However, differences in test sample preparation and in the exposure scenarios prevent clear conclusions from the data. In general, samples were placed in a weathering simulation box for a two-hour exposure to UV or fluorescent light, with a continuous fan and intermittent scraping by a rubber knife. The wood samples were exposed to both the UV and fluorescent lamps, which revealed differences in the rate and concentration of released particles. Compared to UV, exposure to fluorescent light resulted in a slower rate of release and lower concentration of released debris without significantly affecting the median diameter (8.1 – 213.7 nm) of the released particles. Since the authors only used a CPC and SMPS to detect release they were unable to characterize the composition of the released debris.

In a controlled experiment performed by Kaegi et al. [74], release from a product developer’s white acrylic exterior paint containing silver nanoparticles (silver-NP) was analyzed. The authors applied the paint to an expanded polystyrene (EPS) panel with a mineral render base layer and then applied a 2 μm top render with a styrene binder. As before, in a baseline study with pigment grade titania paints [59], a model façade was exposed to ambient weather, which the authors report including 65 rain events. Based on the water samples collected after each rain event, the authors report about 30% of the total amount of silver-NP contained in the paint, or more than 80% of the total amount of silver-NP lost from the paint’s surface, was released during the first two months of weathering, decreasing below the limit of quantification (LOQ) before the end of the experiment. Throughout the experiment, the authors report only observing silver-NP attached to the organic binder under TEM. However, the authors may not have taken into account the probable loss of silver-NP to dissolution and the release of silver ions.

Olabarrieta et al. [75] conducted a controlled experiment to investigate release from coatings containing titania-NP exposed to UV while submerged under water. In this study, the authors used a commercially available paint and a lab-made experimental paint formulated to contain 50% by weight titania-NP (80% anatase, 20% rutile) dispersed in a siliceous matrix. Both paints were coated separately onto thin films (> 600 nm) on glass substrates and submerged in one of four aqueous mixtures while exposed to UV for 1–4 weeks. Based on measurements of total mass loss, atomic force microscopy (AFM) measurement of film thickness and ICP-OES, the authors report both coatings released paint matrix with embedded titania-NP under all test conditions and that the magnitude of release debris was generally higher from the experimental coating. In addition, the authors highlight the potential interactions between the NaCl in solution, UV-A, and titania-NP.

### UV exposure

Several controlled experiments investigating the effects of UV alone on nanocomposites utilized the Simulated Photodegradation via High Energy Radiant Exposure (SPHERE) chamber at the U.S. National Institute of Standards and Technology (NIST). The SPHERE chamber provides a precisely controlled environment (e.g. temperature, humidity) capable of exposing samples composites to continuous UV at desired wavelengths for a desired duration [76]. However, there is currently only one SPHERE chamber. Utilizing this highly specialized accelerated aging SPHERE technology, multiple thermoset nanocomposites with silica-NPs [69-72], and CNTs [70], in addition to a thermoplastic with graphene oxide [68] have been examined. In addition, we were also able to identify an abstract, but no full publication, describing an early study by Pellegrin et al. [77], that reports observed changes in silica-NP concentrations at the surface of amine-cured epoxy nanocomposite films containing 1% and 5% by weight (7 nm) silica-NP prepared by the drawdown technique. The samples were exposed to UV (295 - 400 nm) radiation and the rate of silica-NP release was estimated by thermogravimetric analysis. The authors report rapid degradation of the epoxy matrix, resulting in significant mass loss and gradual increase of surface silica-NP concentration in response to UV treatment. In a follow up experiment by Rabb et al. [72], samples of nanocomposite films containing 0%, 5% and 10% (7 nm) silica-NP in epoxy were placed in the SPHERE for 59 days and examined for surface degradation and silica-NP release through ICP-OES analysis of extracted solutions. The authors report silica-NP on the surface of the nanocomposites and only after 59 days of UV exposure observe increases in the mass fraction of silica-NP on the surface of both the 5% and 10% nanocomposites, but did not detect the release of silica-NP alone.

More recently, Gorham et al. [69] conducted a controlled experiment using a similar amine-cured nanocomposite film with 10% by weight 7 nm silica-NP to investigate release up to 72 days of UV exposure in the SPHERE. As in previous studies, the authors report no direct evidence for the release of dissociated silica-NP, even though the nanocomposite contained no UV stabilizers. The authors clearly demonstrate the accumulation of silica-NP on the surface of the nanocomposite over time, and based on this observation suggest that n-SiO_2_ may eventually release from the degrading matrix. In a separate experiment, Nguyen et al. [71], using similar nanocomposites (5% and 10% by weight) and UV exposure (290-400 nm for 43 days), report under SEM and EDS the first direct evidence that silica-NP or an aggregated form were released from epoxy based nanocomposites during UV irradiation. However, the authors highlight that the preliminary experimental data do not establish whether the released silica-NP are dissociated or embedded within the matrix.

In two separate experiments utilizing the SPHERE, Nguyen and colleagues investigated release of MWCNTs (0.75% by weight) in epoxy nanocomposites [70] and graphene oxide (GO) at 2% by mass fraction in polyethylene terephthalate (PU) sheets [68]. As in previous studies, an amine cured epoxy, commonly used in fiber-reinforced polymer composites, coatings and adhesives, was prepared by drawn down methods with no additional UV stabilizers or additives used. In contrast to the accumulation and eventual release of silica-NP due to polymer surface degradation, the MWCNTs formed a dense layer on the nanocomposite surface by 43 days, which remained even after 9 months of UV exposure. The data show no release of MWCNTs, even after prolonged exposure to intense UV, and the authors suggest that the surface CNT network slows polymer degradation. Similarly, Bernard et al. [68] also report no release of GO from the PU nanocomposite during 137 days of UV exposure. The authors highlight GO accumulation on the nanocomposite surface and suggest PU polymer degradation as the putative mechanism to increasing the surface concentration of GO.

### Standardized weathering methods

Wohlleben et al. [37] were the first to use a standardized ISO weathering method [58] for artificially weathering plastics, and the Suntest™XLS + apparatus to experimentally test several nanocomposites. The nanocomposites included: (1) an author described “worst-case” thermoplastic of polyoxymethylene (POM) containing <5% CNTs with no UV stabilizers, (2) polyamide (PA) compounded with amorphous silica-NP, and two cement mixtures: a (3) cement with a homogenous distribution of CNTs, and (4) cement with 4% synthetic nanoscale calcium silicate hydrates (CSH) nuclei in suspension. In contrast to previous reports with SPHERE [69-72], only minimal PA nanocomposite degradation and no surface accumulation of silica-NP was observed in response to the standardized UV exposure test [37]. In contrast to the PA/silica-NP nanocomposite, the POM-CNT nanocomposite rapidly degraded, exposing CNT fibers on the surface. Wohlleben et al. [37] suggest the rapid surface accumulation of CNTs enhanced UV absorption, further accelerating POM degradation and potential for CNT release, although no release was reported. In contrast to the results with the polymers, the authors reported no differences between cement composites with or without CNTs and no indication of the potential release of the CNTs [37].

In a recent experiment, Wohlleben et al. [37] investigated release from a soft thermoplastic polyurethane with 3% wt added CNTs (TPU/CNT) in response to dry and wet weathering ([57] and [58] respectively). The authors report similar results under both conditions, and similar to other studies [66,67,70]; matrix bound CNTs are exposed on the surface of the degraded polymer, but are not found among the released debris. The authors note however, multiple CNTs protrude from the surface of the degraded nanocomposite, and Hirth et al. [40] report these protrusions can be freed from the matrix by secondary mechanical forces.

Two recently published observations [66,67] used the same ISO accelerated weathering method as Wohlleben et al. [37] on various lab-made nanocomposites. Both of these [66,67] describe complex studies designed to simultaneously investigate: (a) nanocomposite degradation in response to simulated weathering; (b) the effects of nanomaterial modifications on degradation; and (3) the potential for recovery of the added nanomaterials from the nanocomposites through the either acid digestion or calcination.

Vilar et al. [66] used the thermoplastic PA-6 as a base matrix, and added “non-modified” pristine MWCNTs or hydrophilic silica-NPs, or “modified” master batch MWCNTs with 15% nanofiller (PLASTICYL™ PA1503), or hydrophobic silica-NPs with an attached octyl functional group. The authors describe the test nanocomposites with a final 3% nanofiller content without further specification, but according to the Nanocyl company website [78] PLASTICYL™ PA1503 has a 15% CNT content. The test nanocomposites, and PA-6 matrix alone, were exposed to 1000 hours of accelerated weathering following methods [58]. Afterwards, the authors characterized the nanocomposites with a range of techniques (e.g. TGA, TEM, SEM and EDX) but did not collect nor analyze the irrigation water. Thus, release was inferred from data regarding the surface of the weathered nanocomposite, as the released debris would have been in the irrigation water. The TGA data shows loss of organic matter from the weathered PA-6 matrix, both silica-NP nanocomposites, and the non-modified MWCNT nanocomposite but not from the modified MWCNT/PA-6 nanocomposite. In contrast, all nanocomposites exhibited loss of inorganic matter after weathering. Under TEM, the surfaces of all the weathered nanocomposites appear to have a higher concentration of the added nanomaterials. Together, the TGA and TEM data led the authors to suggest that the added nanomaterials were released from all of the nanocomposites during weathering. However, the composition of the released debris could not be determined. In addition, we note that in regards to the modified MWCNT nanocomposite, the TGA and TEM data and the authors’ conclusions appear contradictory. For this nanocomposite, the authors report no loss of organic matter and suggest that the modified MWCNTs may have improved the PA-6 polymer resistance to weathering. Yet under TEM, MWCNTs are clearly visible on the nanocomposite surface. Based on this observation and the loss of inorganic matter, the authors suggest MWCNTs were released. If so, the MWCNT dynamics within the PA-6 matrix would be novel, but since the evidence is indirect and no replicate testing was performed, further investigation is necessary.

In a similar weathering and nanomaterial recovery study, Busquets-Fité et al. [67] investigated lab-made nanocomposites with thermoplastic (PA, PP and ethyl vinyl acetate (EVA)) base matrices, and added 3% wt of silica-NP, titania-NP, zinc oxide-NP, nanoclay or MWCNTs. The authors mention that the added nanomaterials were “functionalized”, but do not describe these in the methods. However, figure legends indicate some of the functionalization; the addition of a propyl group to silica-NP and an octyl group to titania-NP and zinc oxide-NP in the PP matrix based nanocomposites; and the addition of a hydroxyl group to the metal oxide NPs in the PA-6 nanocomposites, and to the MWCNTs in the EVA nanocomposite. All of the nanocomposites were exposed to 1000 hours of weathering following ISO method [58], similar to Vilar et al. [66]. A notable difference between the studies is that Busquets-Fité et al. [67] collected and lyophilized the irrigation water for subsequent, direct analysis. However, the authors provide only a limited description of that analysis, stating that TEM with EDX was used to characterize the release debris but not how the reported mass loss was calculated. The authors report considerable mass loss, roughly 70% from the ZnO_2_-OH/PA-6 nanocomposite, nearly 40% from the TiO_2_-octyl/PP and 20% from the SiO_2_-propyl/PP nanocomposites. Given that only 3% (wt) was the added nanomaterial, it appears considerable degradation of the polymer must have occurred from these nanocomposites, but not the SiO_2_-OH/PA-6, for which both Vilar et al. [66] and Busquets-Fité et al. [67] report nearly identical mass loss. Without replicate testing or controls, we are uncertain what degradation occurred as a result of weathering. Further characterization of the debris released from the SiO_2_-propyl/PP was performed by TEM with EDX. These data led the authors to report silica-NP represented 19.9% of the mass of the released debris, despite only 3% having been added to the nanocomposite. While the authors show release occurred, they do not report the release of dissociated NPs from any of the nanocomposites tested.

Al-Kattan et al. [60] describe experiments investigating release from two paints with identical composition and total titania concentration supplied by industry for this study. The difference between these paints was the titania in one paint comprised only pigment grade (100-300 nm) while the other contained 50% anatase titania-NPs (20-80 nm). The authors investigated release from these paints, applied to large cement fiberboards, in a climate chamber following a European standard for weathering outdoor façades. In an effort to discriminate the potential affects of paint age, aqueous conditions, illumination and the material upon which the paint was applied, the authors also performed a series of laboratory tests with smaller panels. From all experimental conditions, the results are based upon SEM-EDX of weathered paint surfaces and multiple analyses (e.g. ICPMS, ICPOES, TEM) of the leachate produced by irrigation during weathering.

Al-Kattan et al. [60] report both paints, with and without added titania-NPs, released an amount of titania slightly above background levels as a result of simulated weathering. By examining the exposed paint surface under SEM-EDX, the authors report minor degradation of the paint matrix and dispersed, embedded, and agglomerated titania particles ranging from 90-200 nm. In the leachates, the concentration of titania reported by the authors are several orders of magnitude less then reported for pigment grade titania [59] and from silver-NP paints [74] on outdoor façades under natural conditions. However, Al-Kattan et al. [60] were unable to identify any titania in the leachate, preventing analysis of the composition of the released debris. Despite the insignificant release of titania from these paints, the authors varied conditions in laboratory tests on smaller painted panels and dried, aged powders of the paints. Based on these tests, the authors report relative surface area and UV exposure as significantly increasing the release from the titania-NP paint. The paint with only pigment grade titania did not exhibit a similar increase in release. However, considering the lab test results together with the minimal degradation observed under SEM, the authors suggest the UV induced photo-degradation of the paint matrix may not be a simple linear or continuous process, similar to results reported with thermoplastic/CNT nanocomposites exposed to UV [70].

### Aqueous weathering

In an observational study, Zanna et al. [65] investigated the weathering of thin films containing silver-NP applied to steel pipes exposed to saline. The authors prepared two coatings with either a low (7.4%) or high (20.3%) initial concentration of silver-NP and submerged the plasma-coated steel plate samples into saline (0.15 M NaCl), which was stirred and maintained at 30°C to age the coatings for 2, 3, 4, 8, 18 and 60 days. Based on XPS and ToF-SIMS analysis of the nanocomposite films, the authors report very different results for the two types of films tested. The organosilicon nanocomposite of the low silver content film retained its initial thickness, while the thickness of the high silver content film reduced during the immersion time. As such, the authors report oxidation of added nano-silver in the superficial layers of the films. For the low silver content film, the oxidization and release of silver ions from the superficial layers of the film stopped by three days. In contrast, the high silver content film continued to degrade for eighteen days, and thus silver ions were released from the newly superficial layers.

### Weathering summary

**Figure 4:**
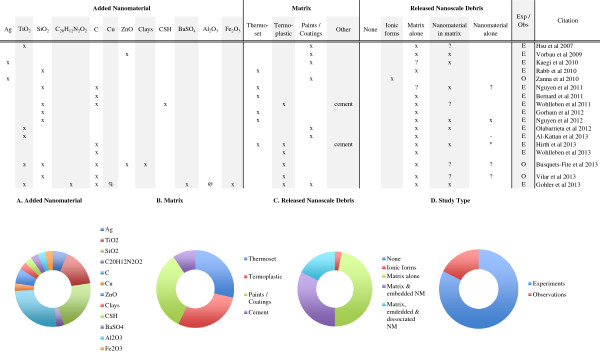
**Weathering induced release from nanocomposites.** About one third of the nanorelease studies have examined release from nanocomposite due to weathering. The most frequently (94%) identified release debris were particles of matrix alone. Particles of matrix with embedded nanomaterial were reported in (65%) of the studies, while about a third (35%) identified dissociated nanomaterials among the released debris from weathered nanocomposites. Across study summaries are present in charts: **(A)** the added nanomaterial, **(B)** the matrix, **(C)** the released debris identified and **(D)** the number of experiments versus observational studies. (*) Authors report release only after a combination of weathering and machining; (-) authors report insignificant but detectable levels of dissociated nanomaterial; (?) data supporting this result are indirect or not presented, but described by the authors; (exp) rigorous experiments with replicate testing and negative controls (samples of matrix without added nanomaterial) examined; (obs) observational studies with no control samples and/or not replicate testing; (C) refers to any one of multiple forms of Carbonaceous nanomaterials, including: single and multi-walled carbon nanotubes, graphene oxide, carbon black and uncharacterized carbon nanotubes; (CSH) are calcium silicate hydrates; (%) a complex copper II with chlorinated phthalocyanine; (@) alumina based Cobalt Blue.

A summary of the seventeen studies examining release from nanocomposites in response to weathering is presented in Figure 4. Among the nanorelease literature, there are more rigorous experiments investigating weathering then any of the other release scenarios. The data highlight the broad range of nanocomposite and matrices tested. While several evaluations were made with specialized equipment (SPHERE), most studies used common laboratory equipment and followed standardized protocols. The released debris was most often the composite alone (94%), but frequently nanomaterials were found embedded within the composite debris as well (65%). Researchers typically relied upon ICP-OES data to indicate that released debris contained both composite matrix and embedded nanomaterial. However, the ICP-OES data is unable to differentiate between nanomaterial particles, or ions of the same element. In addition spectrographic analysis, investigators examined the remaining nanocomposite surface topography by SEM, frequently identifying exposed, bound nanomaterial on the composite’s surface.

Although the data are preliminary, investigators have proposed a model for polymer nanocomposite degradation that occurs around the embedded nanomaterials, which increases with the duration of UV exposure, eventually exposing the nanomaterial [69]. This model for release is nearly identical to one proposed earlier [62] for polymer composites with added, pigment-grade titania. Additional experimentation with nanocomposites would permit researchers to drawn conclusions about polymer degradation and potential nanomaterial release. Indeed, despite multiple reports of added nanomaterials found on the surface of the UV degraded nanocomposites [37,69,70,72,75], Nguyen and colleagues [71] provide the only clear, direct evidence of an added nanomaterial found dissociated from the matrix among released debris. Nonetheless, Al-Kattan et al. [60] report measureable, but insignificant release of dissociated nanomaterials, and two recent studies [66,67] suggest but do not directly show similar release. Thus it is possible that dissociated nanomaterials are released as much as 29% of the time under weathering conditions. And finally, Hirth et al. [40] also clearly identify dissociated CNTs released from the nanocomposite, but only after the combination of machining and weathering.

### Washing scenarios

The potential for release of nanomaterials from nanocomposites during washing may occur in two ways, when the nanocomposite itself is cleaned (e.g. laundering of textiles) or when the nanocomposite is used to clean aqueous solutions (e.g. water filtration). Of these two types of washing, cleaning nanocomposite textiles containing either silver-NP or titania-NP has been investigated most, presumably because of concerns regarding the release of antimicrobials into wastewater treatment facilities, as first raised by Benn and Westerhoff [79] and more broadly by Wijnhoven et al. [80]. As a result, a few investigators have examined the potential for release of silver-NP from a range of laboratory made [81,82] and commercially available textiles [79,83,84] or titania-NP [85] during simulated household washing conditions. In contrast to the washing of nanocomposites, the two studies reviewed here investigate release from nanocomposites used in water filtration. Ren and Smith [86] examined migration and release of silver-NP from ceramic filters produced by various methods, while Bielefeldt et al. [87] investigated the effect water quality has on silver-NP release. We review the washing studies in the following three sections, *novel washing methods, standardized washing methods* and *water filters*.

### Novel washing methods

Benn and Westerhoff [79] were the first to investigate release of silver-NP from commercially available textiles using simulated washing conditions. This observational study was performed once, without controls and highlights a number of challenges associated with investigating a reactive metal that may release ionic species and rapidly form salts. The authors obtained seven pairs of socks from five different manufacturers, and through ICP-OES analysis of one sock from each pair, were able to confirm the presence of silver in six of the seven pairs. Although the authors report a broad range of initial silver concentrations in the socks (0.9 – 1,358.3 µg Ag/g textile), proprietary information about how the silver-NPs were incorporated in the socks were unknown. This information has subsequently been shown to influence nanocomposite stability [81,88]. Nevertheless, each remaining sock from the six pairs that initially contained silver were placed in individual amber bottles with 500 ml of ultra-pure water and agitated on an orbital shaker for at least 3 consecutive “washings” of 1 or 24 hours in duration. Following washing the authors used an ion selective electrode (ISE) to detect silver in the unfiltered wash water, followed by centrifugation and filtration to attempt to identify the proportion of released silver released that was ionic (Ag^+^) versus nanoparticle silver. The authors report three of the six socks leached silver into the wash water at rates of 70-90% of the initial silver concentration, but no correlation between the initial concentration of textile silver and the silver release rate and/or amount. The authors acknowledge multiple confounding variables in their study design, including small sample size, lack of replicates, the use of ultra-pure water and a lack of direct released debris characterization prohibiting further analysis.

Pasricha et al. [82] report a very similar observational study to Benn and Westerhoff [79], but used laboratory made fabrics of cotton, wool and nylon instead of commercially available textiles. In this study, silver-NPs were deposited onto the washed fabrics during an overnight submersion, and subsequently shaken at 600 r/min for 4 hours and dried at 70°C. The test fabrics were then placed in 100 ml glass bottles with 50 ml of ultra-pure water, and either agitated for 60 min using a stirrer or centrifuged at 50 r/min for 30 min. Afterwards the fabrics were wrung out to retain as much wash water in the bottle after each wash cycle, which was repeated five times with new ultra-pure water in each cycle. The five wash cycles for each fabric were repeated three times, yielding considerable data but no control fabrics were tested for comparison. Based on atomic absorption spectrometry (AAS) the authors report that after the first three washes, the cotton fabric released 12% of its silver-NP content. Similarly, the wool fabric released 14% and the nylon fabric released 24%. The authors suggest much of the silver released was in ionic form, but do not elaborate further. They do however highlight that after the five consecutive wash cycle, none of the fabrics released detectable levels of silver, and that the release rates varied among the fabrics. Based on subsequent TEM analysis, the authors report observing colloids of silver with diameters of 8-20 nm, larger then original silver-NPs loaded onto the fabrics. The authors suggest the increase in diameter may be the result of agglomeration, but do not elaborate further or provide additional evidence to determine if the observed particles are salts (e.g. AgCl).

In follow-up observational study with a several of commercially available textiles (e.g. child’s stuffed bear, surgical cloths, medical mask, shirt), Benn et al. [83] address some of the confounding variables identified in their previous work [79], but no control textiles or replicate testing was performed. The authors report each product initially contained detectable levels of silver-NP. The estimated total silver in each of the products was greatest for the medical mask (590 μg) and cloth (810 μg), and yet these products released the least, less than 0.1% of their total silver. In contrast, the shirt, which contained only 44 µg of silver, released nearly 2% of its total silver content - the most among all the products tested. As before, the authors used series filtration, with pore sizes of 450 nm, 100 nm, and 20 nm to measure released debris, and performed a follow-up analysis with SEM to characterize the released particles. The authors report nearly all of the silver released from the child’s toy, towel and the medical products passed through the 20 nm filter. Follow-up SEM of the surface of the medical cloth and face mask, revealed agglomerates of silver-NP with diameters of ~500 nm, coated with nanoparticles <20 nm in diameter. The authors also used EDX analysis on the facemask away from the identified silver-NP agglomerates and identified a dominant silver signal. Based on this signal, the authors suggest the silver passing through the 20 nm filter is either silver-NP and/or ionic silver. In contrast, based on filtration data, two-thirds of the released silver-NP from the shirt was between 100 - 20 nm, and only one-third was less then 20 nm. These findings were only partially supported by observations made under SEM, where agglomerates of 200-500 nm were typically seen in addition to a few particles < 20 nm. The authors suggest that the agglomerates may be an artifact of sample preparation, or that the < 20 nm particles released during washing are not captured by filtration and are actually ionic and not silver-NP.

Geranio et al. [81] conducted an observational textile washing study similar to Benn and Westerhoff [79] but with several key differences in experimental design that permit identification of the form of silver released and influence of pH on release rates. The authors submersed nine different fabrics of known textile composition and method of silver-NP incorporation in a buffered solution of pH 10, with minor agitation (100–150 rpm) for 120 minutes and then added an oxidant to the solution. Under these conditions, all but one of the fabrics released nearly all of its silver content as ionic silver, after the addition of the oxidant, or as particles < 450 nm. The authors suggest these findings are due to the fact that the fabrics contain zero-valent silver-NP, the oxidation of which was required before ionic silver could be detected. In contrast to these alkaline conditions, the silver released under standardized ISO washing conditions ([89] at pH 7) was over 75% as particles > 450 nm, with minimal ionic silver release. The authors suggest this difference was in part due to the mechanical damage caused during the washing process. Geranio et al. [81] highlight a relationship between the amounts of released silver with differences in incorporating the silver-NP in the fabrics. For example, the fabric sample with an electrolytically deposited layer of silver-NP released considerably more silver, while no silver release was detected from the textiles with silver-NP incorporated as a salt (i.e. AgCl). The authors did not perform further analysis of released particles, which may be comprised of silver-NP on fiber fragments, silver-NP aggregates or as AgCl precipitates.

The results reported in Geranio et al. [81] are supported by a recent observational report on release from a broad range of commercially sourced textiles containing silver-NP [84], and experimentally for the first time, titania-NP by Windler et al. [85]. Compared to the controls, the authors clearly observe small agglomerates of titania-NP and AgCl precipitates released from multiple nano-textile composite samples washed under standardized [89] conditions.

### Standardized washing methods

With some modification, Lorenz et al. [84] followed the “color fastness to domestic and commercial laundering” process to simulate laundering of eight commercial textiles containing silver-NP of various compositions and preparations. Although no controls are described, sample textiles were tested with multiple washing and rinse cycles. The wastewater from both the wash and rinse cycles were collected and used to measure released silver-NP by ICP-OES and XRF. Only four of the eight textiles released detectable amounts of silver-NP; these textile samples released 14.8 – 23.5% of their initial silver-NP content as a result of washing. Further characterization reveals that 80% of the released particles from three of the four samples were coarse particulate material >0.45 µm. The authors report observing wash and rinse water containing pure AgCl agglomerates as complex salts (e.g. Si/Ti-AgCl/TiO_2_) or tightly bound agglomerates of metallic silver-NP. However, the release of metallic silver was only observed from one of the samples, and no dissociated silver-NP was observed [84]. The authors conclude that release of silver-NP is unlikely and thus a rare event in contrast to the release of complex particulates. In a separate set of experiments using the same design, Windler et al. [85] examined release from six commercially available textiles that provided UV protection because of the presence of titania-NP (0.22 – 0.85% by weight) in the fabrics. Under the same washing conditions as described by Lorenz et al. [84], the authors report observing only minor (< 0.7 µg/L of Ti) release from five of the six samples, with one sample releasing considerably more (4.7 µg Ti/L) in the washing solution and 0.64 mg Ti/L in the rinsing solution. Of the titania released in the washing solution, 54% was reported to be coarse particulate > 0.45 µm in size. Only two sample fabrics released notable amounts of titania during the course of the experiments, but the authors report all of the released particles to be agglomerates of titania-NP ranging from 60-350 nm in size [85]. Based on these experimental data, the authors draw two conclusions: (1) the release of nanoscale titania from textiles represents, at most, a minor source of environmental titania compared to exterior paints or foods, and (2) that pigment grade titania particles appeared identical (under STEM and TSEM) to added titania-NPs in both the textiles and among the released particles.

### Water filters

One of the most promising point-of-use water treatment technologies available in the developing world today are ceramic-based water filters [90-92]. In the past decade, several groups have enhanced these “low-tech” filters with applications of silver-NP [92-95], which have been shown to effectively remove bacteria [96,97] and protozoan-sized particles [98], thereby disinfecting and clearing water. With over 35 factories in 18 developing countries producing more then 40,000 ceramic filters monthly [92], considerable variability in production exists [86]. How production differences impact the release of silver-NP from ceramic filters [86] and what effects water quality have on release [87] have been addressed in only two studies.

Ren and Smith [86] were the first to investigate release resulting from differences in the methods used to incorporate silver-NPs in ceramic filters. The authors compared ceramic filters with proteinate-capped silver-NPs applied by the “dipping”, “paint-on” and “fire-in” methods. In addition, two amounts 2.76 mg and 27.6 mg (by Ag mass) were applied to each ceramic filter, in an effort to compare the extremes used in production factories in developing countries. The authors then pumped water through each filter at a flow rate of 0.6 mL/min for three hours and then increased to 1.2 mL/min for another three hours. Effluent water was collected and silver content was measured by ISE. The authors report 1.1 - 1.3% silver release from the paint-on and dipping method filters, respectively. But do not report the original amount of silver-NP added, either 2.76 mg or 27.6 mg. In contrast, the authors report release for both original silver-NP concentrations from the fire-in produced filters. The filter with 27.6 mg silver-NP released a smaller percentage then the 2.76 mg silver-NP filter, and both release significantly less (0.001%) than filters produced by the other two methods. While further investigation is necessary, these data suggest that production methods may significantly affect the release of silver from ceramic-based filters.

Bielefeldt et al. [87] were the first to investigate the effects of varying water qualities (e.g. pH, turbidity, dissolved organic mater) on the potential for release of silver-NP from ceramic filters. The authors used commercially available silver-NP suspended in casein and followed Nardo [94] to prepare a 0.018% silver-NP solution with ultra-pure water. The authors then deposited the silver-NP solution on to silica-coated sensors amenable to the quartz crystal microbalance (QCM) technique. The QCM technique [99] allows measurement of the mass, viscosity and density of a sample, in this case silver-NP, at the surface of the sensor. Thus, the authors were able to investigate the behavior of silver on the silica filter rather then inferring release by monitoring affluent water content or changes in disinfection efficiency. In this experiment, the authors performed multiple baseline tests using sensors with and without silica coatings, and on silica-coated sensors without silver-NP. These baseline data could be compared to the measurements obtained over multiple two-hour experiments using the silica-coated sensors with deposited silver-NP, which represent the silver-NP impregnated ceramic filters. Based on the QCM data, the authors report minimal silver-NP release from the silica-coated sensors under typical ranges of pH, turbidity, ionic strength, dissolved organic matter. However, the authors also report significant release occurs under more extreme conditions, with a 20% mass loss in to ultra-pure water, a 21% loss in the water with a total organic content of 15 mg/L, a 24% loss in low pH (4.8) and as much as 86% mass loss of silver in the presence of even small amounts of bleach (NaOCl). On the QCM data alone, the authors are unable to determine if the mass loss of silver is due to the release of silver-NP or silver ions, but based on TEM the authors suggest the loss is most likely the result of enhanced dissolution of silver ions.

### Washing summary

**Figure 5:**
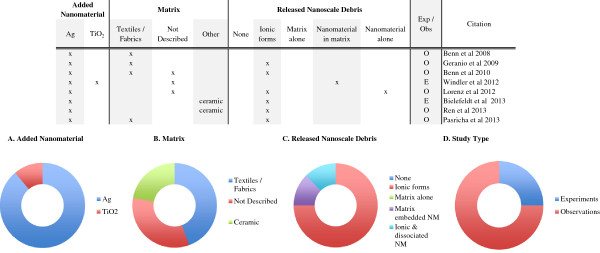
**Washing induced release from nanocomposites.** While only eight studies have focused on release during washing, either of the nanocomposite or through the use of the nanocomposite as a filter, all examined the release of silver-NPs. The data presented in these studies strongly indicates release of ionic forms of silver (75%), but at this time it is difficult to determine if these data are accurate, or limited by the methods used to detect release in most studies. One study shows the release of the matrix with embedded nanomaterials, and one clearly demonstrates release of both the ionic form and dissociated nanoparticulate silver. Across study summaries are present in charts: **(A)** the added nanomaterial, **(B)** the matrix, **(C)** the released debris identified and **(D)** the number of experiments versus observational studies. (-/+) authors report minimal but detectable levels of dissociated nanomaterial under normal conditions, but significant release under extreme conditions; (exp) rigorous experiments with replicate testing and negative controls (samples of matrix without added nanomaterial) examined; (obs) observational studies with no control samples and/or not replicate testing.

The market availability of textiles and filters that contain nanomaterials with anti-microbial properties has drawn attention among researchers to investigate nanomaterial release during and after washing these products. A summary of the eight observational studies and two experiments examining release due to washing is presented in Figure 5. The data highlight the narrow focus on silver-NPs added most frequently to textiles or ceramic filters, and one study investigating titania-NPs. Unfortunately, many of these studies do not provide any description of the textile nanocomposite, thus limiting readers’ ability to interpret the results. In addition, much of the data about release was through ICP-OES and/or ISE, and authors frequently reported the release of ionic forms of the added nanomaterial, but are unable to identify nanoparticles. Multiple reports highlight the challenge of detecting release of silver-NP in an aqueous environment before it forms a salt, and in distinguishing that event from the release of a salt or silver ions. Only Lorenz et al. [84] potentially demonstrate the release of metallic silver-NP, but even in this paper the authors note that the majority of release occurs as salts or ions, and not as nanoparticles.

Regarding the two studies investigating release from ceramic water filters impregnated with silver-NPs, the data show considerable potential differences in release due to production methods and water quality. While such filters offer tremendous potential to deliver clean, safe water at very low cost, further study is likely warranted given the application. However, these are the only release studies we are aware of examining water filters, despite multiple commercially available water filters utilizing nanomaterials.

### Contact scenarios

Some nanocomposites are designed to come into direct contact with humans including, dental fillings [55], medical devices [100-102], and textiles [103,104]. Also, nanocomposites designed for vehicle exteriors have been examined for release in the context of collisions [105]. While the work of Moreau et al. [55] with dental composites was discussed in the machining section, here we review four other studies in the following three sections, *standardized sweat methods*, *medical applications* and *collision.*

### Standardized sweat methods

Kulthong et al. [103] describe the first investigation of silver-NP release as a result of exposure to sweat, instead of washing. The authors used standardized artificial human sweat applied to fabrics with known silver-NP loads and commercially available fabrics with unknown silver-NP loads. In addition, the authors tested the bactericidal strength of these fabrics on both Gram-negative and Gram-positive bacteria. In this study, five cotton fabric samples were coated with known concentrations of silver-NP in the laboratory, and six commercially available fabrics were obtained from different manufactures in Thailand claiming to use “nano-silver” in their product. The initial silver content in the laboratory prepared samples correlated closely with the known amount of silver coated onto the fabric (0 mg/kg – 500 mg/kg). Meanwhile, half of the commercial fabrics were reported to have no detectable silver. The remaining three commercially available shirts initially contained as much as 20 mg/kg to as little as 1 mg/kg of fabric. Interestingly, despite the low levels of detected silver-NP in the commercial fabrics, they were just as effective with respect to bactericidal properties as the lab prepared fabrics to Gram-positive bacteria and were more effective to the Gram-negative bacteria.

To assess the release of silver-NP from fabrics immersed in artificial sweat, Kulthong et al. [103] used standardized sweat formulations ([106-108]). Two different ISO formulations were prepared at two different pH levels, 5.5 and 8.0. Because of the low concentration of silver in the commercial fabrics, these were soaked in artificial sweat at a ratio of 1:50 (w/v), while the lab prepared samples were soaked at a ratio of 1:100 (w/v). Samples were incubated in each of the four standardized artificial sweat formulations for 24 hours at 37°C, and release was analyzed by graphite furnace atomic absorption spectroscopy (GFAAS). The laboratory prepared samples released silver-NP (except the negative control) in response to incubation in all of the sweat formulations. Comparisons between the two pH levels used in the ISO formulation indicate that a more alkaline pH results in greater release. In contrast to the lab prepared samples, there was variable release from the commercial fabrics that contained silver. One did not appear to release any silver under any of the conditions. Another released a minimal amount in the EN sweat formulation only. The third released minor amounts of silver-NP in response to every sweat formulation. Although the authors report the release of silver-NP, the method used to detect released silver, GFAAS is an elemental analysis, unable to distinguish among the various forms of silver.

In a similar study, von Goetz et al. [104] examined the release of both silver-NPs and titania-NPs from a range of commercially available textiles exposed to both artificial sweat and physical stress. Although control fabrics were not tested, the authors made considerable effort to use standardized methods (ISO 105-E04), a reproducible experimental design and avoided unrealistic conditions (e.g. temperature, excess artificial sweat), but also attempted to create a ‘worst-case’ scenario. These considerations increase the value of both the data and the approach used. In this study, the authors obtained nine sports apparel textiles whose manufacturers claimed contained either silver-NPs, titania-NPs or in one fabric, contained both. The initial content of silver-NP and titania-NP was confirmed by the authors, and revealed a 1000-fold higher initial concentration of titania-NP in the textiles. To induce release, the authors used 8 g of each textile as a test fabric and followed a modified “color fastness to domestic and commercial laundering” standard method (ISO 105-C06). The modifications include using artificial sweat (ISO 105-E04), and adding this solution to polyethylene bottles and replaced the steel balls, used to simulate physical stress, with acrylic plastic ball to, both to reduce background silver levels. Although the authors tested various exposure times, 30 minutes (in 120 ml of artificial sweat at 40°C) of agitation was determined to be the minimum necessary for reliably repeatable experiments. Fractional filtration of the artificial sweat after incubation was used to isolate particulate silver and titania released form the test fabrics. Based on ICP-OES analysis, the authors report that seven of the nine textiles examined released no detectable levels of either silver-NP or titania-NP. Test fabric from textile #2, which contained both silver-NP and titania-NP released dissolved and particulate silver, and particulate titania under both acidic and alkaline sweat conditions. In addition, textile #4 released dissolved silver in response to both acidic and alkaline sweat, as well as particulate silver <450 nm under alkaline conditions. Subsequent STEM analysis of the filtrate from textile #2 revealed agglomerates of titania-NPs of 150-300 nm and silver containing particles of 20-200 nm. Further EDX analysis of the leachate from textiles #2 showed the particles contained silver and chloride, suggesting rapid salt (i.e. AgCl) formation after due to the high chlorine content of the artificial sweat. In contrast, the leachate from textile #4 observed under STEM contained no detectable particles at all. The authors then compare the total released silver from textiles #2 and #4 in acidic/alkaline sweat (5%/7% and 13%/14%) in this study to the maximum release (3.3%) reported by Kulthong et al. [103], and suggest that physical stress may lead to in increased silver release rates. However, the number of factors involved in textile fabrication confounds further comparisons.

### Medical applications

In addition to fabrics, manufacturers have begun to incorporate silver-NP particles into the polymers used to form catheters in an effort to minimize infections. Although these state-of-the-art antimicrobial polymer catheters were presumed to release silver ions continuously [100-102]; Joyce-Wöhrmann et al. [109] were the first to directly investigate release. In a brief communication, the authors report their observation that silver ions were released from thermoplastic polyurethane catheters with varying amounts of added silver-NP after a 24 hour incubation in physiological saline held at 37°C. While the group reports dose dependent release of silver ions from the catheters, they did not characterize the dynamics of the release in more detail.

### Collision

Another potential use for polymer nanocomposites is in structural applications that absorb impact in a controlled manner. Sachse et al. [105] are the first to investigate the potential for release from nanocomposites as the result of impact. In this novel experiment, the authors made a series of crash cones of thermoplastic polymers (PP and PA-6) alone, and with either silica-NPs or nanoclays added. Cones were crushed by dropping a 54 kg mass impactor at three different velocities (4.4, 6.2 and 7.7 m/s) resulting in impact forces of 520, 1050 and 1580 joules, respectively. Two experiments with each type of crush cone were performed within a crash chamber, to which a CPC and SMPS were connected and used to detect debris released during impact. Under these test conditions, the authors report both matrices (without nanomaterials) released debris when crushed, but only report the nanoscale particle size from the PP composite (15 – 60 nm) cones. Similarly, the authors describe the PA-6 and PP/nanoclay nanocomposites releasing the most nanoscale particles during impact, but do not provide the supporting data. The only data provided relates to the PP control matrix and the PP/silica-NP nanocomposite. At the low input energy and highest input energy impacts, the nanocomposite releases fewer nanoscale particles when compared to the PP matrix. However, at the mid-impact energy (~1000 J) the nanocomposite releases more then the matrix, indicating some potentially interesting and, as yet, poorly understood material dynamics. Given the potential applications of nanomaterial enhanced polymers being used to absorb impact (e.g. vehicles), this is a valuable release scenario that merits further investigation.

### Contact summary

**Figure 6:**
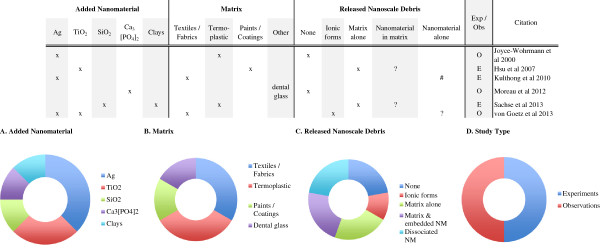
Contact induced release from nanocomposites. Studies examining release from a nanocomposite in either direct contact with humans, or in a collision scenario, report a range of results. Two studies report no detected release. Two studies report detecting release of the matrix with and without embedded nanomaterials. And two studies report the release of the nanomaterial dissociated from the matrix. One other these studies used a detection method (GFAAS), which destroys the sample material in the process, so it is impossible to determine if the nanomaterial was dissociated or attached to the matrix. The other study used ICP-OES to indirectly show the release of the nanomaterial in addition to ionic forms. Across study summaries are present in charts: **(A)** the added nanomaterial, **(B)** the matrix, **(C)** the released debris identified and **(D)** the number of experiments versus observational studies. (?) data supporting this result are indirect or not presented, but described by the authors; (exp) rigorous experiments with replicate testing and negative controls (samples of matrix without added nanomaterial) examined; (obs) observational studies with no control samples and/or not replicate testing; (#) Authors used GFAAS, which incinerates the matrix in the process of identifying chemical composition. Thus we are unable to determine if the nanomaterial was bound to, or dissociated from the matrix.

While we are aware of only six studies in this category, these have examined a relatively wide range of nanomaterials, matrices and contact scenarios (Figure 6). Two report no release, two report release of the matrix alone and matrix with embedded nanomaterials, and two report the potential release of dissociated nanomaterials [103,104]. These data are preliminary, but raise interesting questions about potential release from nanocomposites when in direct contact with humans, or during collisions. More investigation is certainly warranted in these scenarios.

### Incineration scenario

Due to manufacturer and consumer concern about the potential flammability of many polymers, on-going efforts to develop composites resistant to combustion [110,111] have recently focused on the potential utility of nano-clays [112-117], carbon nanotubes [113,118-121], graphite [122] and titania-NP [123] as flame retardants. Three of these efforts [113,122,123] considered release indirectly, and all reported observing a thick layer of the added nanomaterial near the surface of the remaining char. Compared to char from conventional composites, the nanocomposite char exhibited increased stability; leading authors to suggest that addition of some nanomaterials may enhance the composite’s protection against thermo-degradation [113,122,123]. These observations, like others [2-4] demonstrate increased composite durability due to the addition of specific nanomaterials, are encouraging signs of material improvements with direct consumer benefit.

More recently published studies have focused directly on understanding release from nanocomposites, resulting from combustion [124,125] and specifically at the end of a product’s life cycle during waste incineration [126]. Similar to previous observational studies, Uddin and Nyden [125] reported identifying the CNFs that had been added to polyurethane foam (PUF) in the remaining char and not in the aerosolized soot. The authors hypothesized that any CNFs released from the PUF were immediately destroyed during the combustion. However, the lack of equipment capable of capturing and characterizing released nanomaterials during combustion remains a significant challenge in the field [126].

Motzkus et al. [124] developed a novel setup to investigate nanoscale debris release during incineration of nanocomposites. The authors report on a combustion chamber that has been fitted with a condensation nuclei counter (CNC) coupled with a cone calorimeter to measure the released particle mass distribution and concentration within the aerosolized soot following ISO methods [127]. Subsequent examination with an AFM permits characterization of the release debris composition. Using this setup, the authors are the first to experimentally investigate release from multiple nanocomposites. The authors report on release from nanocomposites made with one of three nanomaterials (silica, alumina and CNTs) added to one of two different thermoplastic matrices (PMMA and PA-6), and in one nanocomposite a flame-retardant (ammonium-phosphate (APP)) is added. These data are compared to the debris released from the matrix polymers (PMMA and PA-6) alone. Unfortunately, the authors describe only a subset of their results. Based on the data presented and results described, the authors show detectable release of submicron and ultrafine particles during combustion of both PMMA and PA-6 alone. The addition of nanomaterials, silica-NP and alumina-NP to PMMA significantly reduces the rate and concentration of submicron particles released during incineration. Under AFM, the authors report that nanoparticles are visible on the surface of PMMA sample and putatively in increased number on PMMA/alumina-NP with APP.

Bouillard et al. [126] developed another novel experimental set up to address the challenge of identifying and capturing potentially released nanomaterials in the aerosolized soot. The authors utilized an EPLI to measure particle size and distribution, and captured release particles on an aspiration-based TEM grid for subsequent characterization. The authors selected a polymer matrix commonly used in the automobile industry, acrylonitrile butadiene styrene (ABS) extruded with MWCNTs at 3% (wt) in the final thermoplastic nanocomposite. During incineration of a control matrix (without CNTs) and the nanocomposite, the authors reported detecting nanoscale carbon particles 10-30 nm in size. In addition to the released primary carbon particles, the authors detected single and bundled MWCNTs in the aerosolized soot from the nanocomposite. The authors report identifying MWCNTs roughly 12 nm in diameter and 600 nm in length, similar in size to the MWCNTs originally added to the nanocomposite. These are the first and currently the only data to show release of an added nanomaterial from a nanocomposite during incineration. Furthermore, this study may also present a methodological approach that can successfully detect release that may have been previously missed.

### Incineration summary

**Figure 7:**
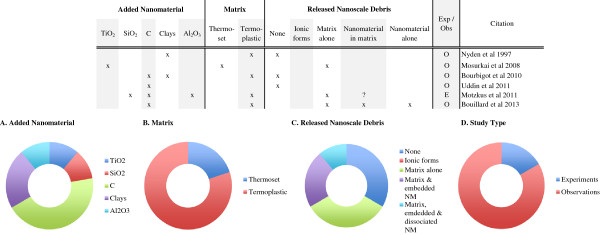
Incineration induced release from nanocomposites. The incineration of nanocomposites could occur accidentally, or as part of disposal of a nanocomposite product at the end of its life cycle. Despite the importance of these scenarios, few studies have investigated release due to incineration. Early investigations reported no release, or release of the matrix alone. In the most recent investigation, release of the matrix, matrix embedded with the nanomaterial and dissociated nanomaterial were all reported. Across study summaries are present in charts: **(A)** the added nanomaterial, **(B)** the matrix, **(C)** the released debris identified and **(D)** the number of experiments versus observational studies. (?) data supporting this result are indirect or not presented, but described by the authors; (exp) rigorous experiments with replicate testing and negative controls (samples of matrix without added nanomaterial) examined; (obs) observational studies with no control samples and/or not replicate testing; (C) refers to any one of multiple forms of Carbonaceous nanomaterials, including: single and multi-walled carbon nanotubes, graphene oxide, carbon black and uncharacterized carbon nanotubes; (CSH) are calcium silicate hydrates.

A summary of the six incineration studies is presented in Figure 7. All but one of these studies have been observational in nature, most have focused on CNTs added to thermoplastics and in only one study has the release of the added nanomaterial been identified. While several studies have demonstrated the increased stability of nanocomposites during combustion, these were unable to address the potential release. Indeed, as pointed out by Bouillard et al. [126] significant challenges exist when attempting to distinguish among the released nanoscale particles during combustion. Continuing efforts in this are may begin to elucidate the actual potential for release from nanocomposites during incineration.

### Nanoscale particle release from conventional products

A topic of considerable importance when considering any novel risks associated with nanocomposites is the release of nanoscale particles from conventional composites and bulk materials exposed to similar processes. Although there is extensive literature on the release of ultra-fine and fine particles from a wide range of anthropogenic combustion (e.g. waste treatment and traffic-related) and non-combustion (e.g. agriculture, break and tire wear) activities (reviewed by [128]), we chose to highlight only two relevant studies here for the sake of brevity.

In a study frequently cited as investigating release from a nanocomposite, Kaegi et al. [59] investigated release of nanoscale titania from a conventional paint containing only pigment grade titania. While this study is novel in approach and the first we are aware of that highlights the potential for titania-NP release from conventional paints, where up to 30% of the pigment grade titania may be in the nanoscale [129], the paint examined was technically not a nanocomposite. In this study, the authors compared rainwater runoff from two painted sources, an ‘aged’ façade and a ‘new’ model façade, and compared two sites of rainwater collection, directly beneath the facades and some unknown distance away from these facades in what is described as an ‘urban catchment’. After rain events, the authors used series centrifugation to remove the larger particles from the collected water samples and then performed chemical analysis with inductive coupled plasma mass spectrometry (ICP-MS) and ICP-OES. The authors report identifying released debris of paint organic binder with embedded titania-NP particles in the runoff from both the new and aged façades. Further analysis, with SEM and TEM of the samples from both façades revealed individual and clumped titania-NP nanoparticles partially embedded in the matrix with diameters of roughly 150 nm. The authors report being unable to identify any discrete titania-NP particles dissociated from the organic paint matrix in the runoff water directly beneath either façade, but do report dissociated titania-NP and agglomerates in the urban runoff. Given the distance from the façades and the inclusion of runoff from other urban structures, the source of the titania-NP is unknown and may have been released from a paint, or could be naturally occurring [130].

In another study, Vargas et al. [131] investigated release of nanoscale copper from standard, bulk copper plumbing pipes during ageing and repeated flushing. The copper plumbing used in this study is the same conventional plumbing pipe used in nearly all homes and commercial buildings in North America, Europe and elsewhere. With no added nanomaterial in the pipe, the authors report copper-NP release from aged plumbing after repeated flushing. The authors suggest release could have been in the form of, (1) cupric ions released from cuprous oxide (Cu_2_O) oxidation, (2) Cu^2+^ due to the solubility of solid corrosion by-products, or (3) particulate copper-NP detached from the pipe. Under SEM, the pipe’s interior appeared to be coated by a film of corrosion by-product, which the authors suggest formed by the aggregation of structures with a size < 2 nm. These data are novel, and suggest the possibility that nanoparticles of metals may release from bulk metallic sources under specific conditions.

In a final note on the topic of nanoparticle release, throughout this review we discuss studies that used control composites and base matrices (without any added nanomaterial) where the authors consistently report identifying the release of nanoscale debris. As with conventional composites and bulk materials, nanoscale particles are often released, highlighting the pedestrian nature of nanoparticle release under natural and mechanical processes.

### Overall summary

We review fifty-four studies that investigate release from solid, non-food, nanocomposites. These early efforts to understand release from nanocomposites, examined a variety of materials and methods under five general scenarios – machining, weathering, washing, contact and incineration. Some of studies (e.g. [37,68-70]) used “worst-case” nanocomposites so that the authors could focus on validating the methodologies used to induce release and/or detect and characterize the debris. Other studies selected commercially available nanocomposites based on manufacturer claims (e.g. [79,83-85]), or were provided test nanocomposites by industry (e.g. [26,29,34]) for the purpose of evaluating release. And several studies examined lab-made, frequently novel nanocomposites, which may have little or no relevance to commercially viable nanocomposites. The differences in the nanocomposites, along with the lack of harmonized methods means the current nanorelease data cannot be readily compared. Furthermore, these data might not necessarily be indicative or predictive of release from commercial nanocomposites in general. Rather, these data demonstrate what types of release particles one might expect, and highlight the methodological approaches that, to varying degrees, can be used to induce release and to analyze the resulting debris.

### Release debris

**Figure 8:**
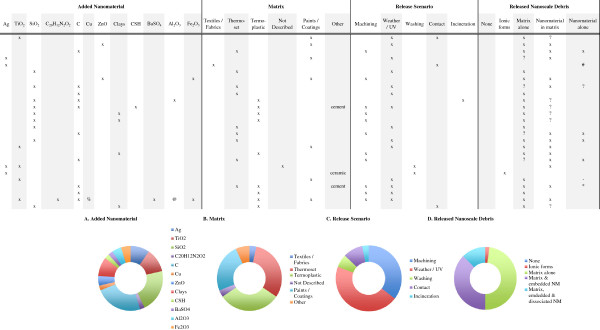
**Summary of release from experimentally examined nanocomposites.** Half of the nanorelease studies published to date are rigorous experiments that include, negative controls (samples of matrix without added nanomaterial) and repeated testing. While the observation studies are informative, these experiments provide the best current evidence about release from nanocomposites. As with the nanorelease studies in general, most of the experiments have examined machining and weathering scenarios. In addition, authors have tended to focus on nanocomposites of either thermoset or thermoplastic matrices, with the addition of either silica-NP or some carbonaceous structure. In all of these studies, authors report detecting some type of released debris. In 69% of these studies, the authors a mixture of release debris types; 88% identified matrix debris, 73% identified nanomaterial embedded within matrix particles and 31% identified dissociated nanomaterial. Across study summaries are present in charts: **(A)** the added nanomaterial, **(B)** the matrix, **(C)** the release scenario examined, **(D)** the released debris identified. (#) Authors used GFAAS, which incinerates the matrix in the process of identifying chemical composition. Thus we are unable to determine if the nanomaterial was bound to, or dissociated from the matrix; (*) Authors report release only after a combination of weathering and machining; (-) authors report insignificant but detectable levels of dissociated nanomaterial; (?) data supporting this result are indirect or not presented, but described by the authors; (C) refers to any one of multiple forms of Carbonaceous nanomaterials, including: single and multi-walled carbon nanotubes, graphene oxide, carbon black and uncharacterized carbon nanotubes; (CSH) are calcium silicate hydrates; (%) a complex copper II with chlorinated phthalocyanine; (@) alumina based Cobalt Blue.

While keeping the limitations of these studies in mind, a summary of the experimental data that has been reported to date (Figure 8) yields some insights. It is immediately clear that at least some nanoscale debris was frequently released from nearly all of the nanocomposites (96% of the experimental studies) and base matrices examined. In the experimental studies where release occurred, matrix particles were almost always detected (92%), and nanomaterials partially embedded within matrix particles were frequently detected (76%). This frequency may in fact be higher, because fully embedded nanomaterials may go undetected due to the limitations of some methodologies used. Finally, the release of dissociated nanomaterials was only detected in 31% of the studies in which release occurred.

While definitive conclusions should not be made at this time, the data suggest two likely trends. First, many of the detection methods are effective and they show release to be frequent but limited in volume. Second, the frequent release of matrix with embedded nanomaterials along with the infrequent release of dissociated nanomaterials implies that while degradation occurs, nanomaterials often remain tightly bound to the matrix. In studies where this dynamic was rigorously examined, tight binding between added nanomaterials and matrix have been reported [37,39,60,70]. However, far more investigation will be necessary to elucidate the conditions affecting nanomaterial-matrix binding. At this time, a wide range of nanomaterials have been added to a few base matrices to form nanocomposites, but generally speaking only a few of the same nanocomposites have been examined multiple times. A silica-NP/epoxy nanocomposite has been examined under weathering [69-72] and a set of nanocomposites with cement or thermoplastic matrices (POM or TPU) with embedded CNTs [37,39-41] have been weathered, machined or both. The results from these studies show that release of matrix particles with and without embedded nanomaterials is common, but the release of dissociated nanomaterials is rare.

### Release methods

In addition to the materials and results of these studies, we were interested in the methods used to induce release. In general, there are no ideal methodologies in use and we agree with suggestions that harmonization would be beneficial [19]. Of the release scenarios examined most frequently (machining and weathering) both novel and standardized methods have been employed with varying degrees of success.

Machining forces were applied through several non-standardized and often hand-held devises, to cut [25,26,45], grind [26,27], shred [28] sand [19,29,34,36-39] and drill [43-45]. The only standardized equipment used in machining studies is the Taber Abraser [37,39,42,46-50]. However, how any of these machining forces relate to real-world conditions remains largely unknown. Given the increasing interest in adding nanomaterials to construction composites and coatings, additional research on potential release from these materials will be important to further understanding the exposure potential to nanomaterials.

The effects of long term weathering on nanomaterial release will also be important to understand, as new structures are built or resurfaced with nanocomposites. Many weathering studies relied upon UV exposure alone, either in the unique SPHERE [68-72], by following ISO methods [37,39,57,58,66,67] or European standards [60]. We believe standardized methods are useful, but given the proportion of the world’s population living along maritime coasts, perhaps considering salinity would also be helpful.

The remaining release scenarios have been investigated so little that it is not known what methods or standards might be most valuable. In addition to encouraging more investigation into all of the release scenarios, it may be important to consider examining other release scenarios, such as during bio-fouling. Nanocomposites capable of either inhibiting bio-fouling or exhibiting ‘self-cleaning’ properties, could be attractive coatings for use on ship hulls, buildings, automobiles, shipping containers, and grain elevators or hoppers. Despite this broad range of potentially beneficial applications and, in some cases, the direct contact such nanocomposites would have with materials and bulk commodities, we were unable to identify any nanorelease studies that investigated such a scenario.

### Overall perspective and future directions

This review demonstrates the relative shortage of research into the release of manufactured nanomaterials in processes that model commercial use to date. The work that has been done in this area, while based on sound science, has not been done in a consistent fashion making the results challenging to put into perspective across nanomaterials, matrices and release scenarios. In addition, the instrumentation used to determine release is highly variable, and this too poses a major challenge for future work. The field will benefit from increased attention on a limited set of high priority nanomaterials, matrices, methods, and instrumentation that will allow for results that can be compared to each other leading to meaningful information about the release potential of nanomaterials. We believe this will build a better foundation for work that will help contribute to a more consistent understanding of the release potential of nanomaterials, which can be translated into potential exposure data that can help to inform risk assessments. Importantly, the robust nanorelease evaluations conducted to date do not indicate a high propensity for discreet nanomaterial release, but rather composite particles of matrix with partially or fully embedded nanomaterials. While far more research is necessary, these data are a good starting point for consideration of potential consumer or environmental exposure to the actual released material from nanocomposites.

## Abbreviations

AAS: Atomic absorption spectrometry; AATCC: American Association of Textile Chemists and Colorists; AFM: Atomic force microscopy; AgCl: Silver chloride; APP: Ammonium-phosphate; APS: Aerosol particle sizer; ASTM: American Society for Testing and Materials; Ag+: Ionic silver; BS: British Standard; CNC: Condensation nuclei counter; CNT(s): Carbon nanotube(s); CPC: Condensation particle counter; CSH: Calcium silicate hydrates; EDX: Energy dispersive X-ray spectroscopy; EEPS: Engine exhaust particle sizer; ELPI: Electrical low pressure impactor; EPS: Expanded polystyrene; EPA: U.S. Environmental Protection Agency; FMPS: Fast mobility particle scanner; GO: Graphene oxide; GFAAS: Graphite furnace atomic absorption spectroscopy; ICP-MS: Inductive coupled plasma mass spectrometry; ICP-OES: Inductively coupled plasma optical emission spectroscopy; ISE: Ion selective electrode; ISO: International Standards Organization; LAP: Laser aerosol particle sizer; LOQ: Limit of quantification; MDF: Medium density fiberboard; mg: Milligram; µm: Micrometer; MMT: Montmorillonite nanoclay; MWCNT: Multi-Walled Carbon Nanotube; NaCl: Sodium chloride; NAS: Nanometer aerosol sampler; NCBI: National Center for Biotechnology Information; NIST: U.S. National Institute of Standards and Technology; nm: Nanometer; NP: Nanoparticle; OPC: Optical particle counter; PA: Polyamide; PC: Polycarbonate; PET: Polyethylene terephalate; PMMA: Poly (methyl methacrylate); POM: Polyoxymethylene; PU: Polyurethane; PVC: Polyvinyl chloride; QCM: Quartz crystal microbalance; SEM: Scanning electron microscopy; SIMS: Secondary ion mass spectrometry; SMPS: Scanning mobility particle sizer; SPHERE: Simulated photodegradation via high energy radiant exposure; STEM: Scanning transmission electron microscopy; TCLP: Toxicity characterization leaching procedure; TEM: Transmission electron microscopy; TGA: Thermogravimetric analysis; ToF SIMS: Time of flight secondary ion mass spectrometry; TSEM: Transmission mode scanning electron microscopy; UV: Ultraviolet light; wt: Weight; w/v: Weight to volume ratio; XPS: X-ray photoelectron spectroscopy; XRF: X-ray Fluorescence.

## Competing interests

The authors declare that they have no competing interests.

## Authors’ contributions

All authors participated in a preliminary discussion of the literature search and scope. SJF and SFC conducted the literature search. SJF reviewed, summarized and analyzed the literature; developed the conceptual framework to present the analysis; formulated the key summary points of the review and drafted the manuscript and figures. SFC and DRB assisted SJF with the development of the manuscript outline and final scope; performed review and interpretation of data, tables, and figures; and supported the preparation of the final manuscript. All authors approved the final manuscript.
